# Mathematical Modeling Predicts that Increased HSV-2 Shedding in HIV-1 Infected Persons Is Due to Poor Immunologic Control in Ganglia and Genital Mucosa

**DOI:** 10.1371/journal.pone.0155124

**Published:** 2016-06-10

**Authors:** Joshua T. Schiffer, David A. Swan, Amalia Magaret, Timothy W. Schacker, Anna Wald, Lawrence Corey

**Affiliations:** 1 Vaccine and Infectious Diseases Division, Fred Hutchinson Cancer Research Center, Seattle, Washington, United States of America; 2 Clinical Research Division, Fred Hutchinson Cancer Research Center, Seattle, Washington, United States of America; 3 Department of Medicine, University of Washington, Seattle, Washington, United States of America; 4 Department of Laboratory Medicine, University of Washington, Seattle, Washington, United States of America; 5 Department of Biostatistics, University of Washington, Seattle, Washington, United States of America; 6 Department of Medicine, University of Minnesota, Minneapolis, Minnesota, United States of America; 7 Department of Epidemiology, University of Washington, Seattle, Washington, United States of America; The University of Melbourne, AUSTRALIA

## Abstract

A signature feature of HIV infection is poor control of herpes virus infections, which reactivate from latency and cause opportunistic infections. While the general mechanism underlying this observation is deficient CD4+T-cell function, it is unknown whether increased severity of herpes virus infections is due primarily to poor immune control in latent or lytic sites of infection, or whether CD4+ immunodeficiency leads to more critical downstream deficits in humoral or cell-mediated immunologic responses. Here we compare genital shedding patterns of herpes simplex virus-2 (HSV-2) in 98 HIV infected and 98 HIV uninfected men matched on length of infection, HSV-1 serostatus and nationality. We demonstrate that high copy HSV-2 shedding is more frequent in HIV positive men, particularly in participants with CD4+ T-cell count <200/μL. Genital shedding is more frequent due to higher rate of shedding episodes, as well as a higher proportion of prolonged shedding episodes. Peak episode viral load was not found to differ between HIV infected and uninfected participants regardless of CD4+ T-cell count. We simulate a mathematical model which recapitulates these findings and identifies that rate of HSV-2 release from neural tissue increases, duration of mucosal cytolytic immune protection decreases, and cell-free viral lifespan increases in HIV infected participants. These results suggest that increased HSV-2 shedding in HIV infected persons may be caused by impaired immune function in both latent and lytic tissue compartments, with deficits in clearance of HSV-2 infected cells and extracellular virus.

## Introduction

Herpes simplex virus-2 (HSV-2) is characterized by frequent, highly heterogeneous episodes of genital viral shedding.[1, 2] The most common type of episodes last only a few hours, are associated with low peak viral loads (<10^3^ HSV DNA copes) and are usually not symptomatic.[3] Yet, many infected people also have occasional episodes that last more than a week, have higher viral loads (>10^7^ HSV DNA copies), and result in crops of genital ulcers and vesicles that are the signature feature of infection.[4–6]

Most immunocompetent people ultimately contain even these more prolonged episodes, and systemic involvement of infection is uncommon, implying a robust albeit not fully protective level of mucosal immunity against the virus. Persons with HIV infection, particularly those with low circulating CD4+ T-cell counts, are sometimes unable to control genital HSV-2 and can develop severe persistent lesions despite the use of potent antiviral therapy.[7, 8] Ultimately, drug resistant HSV-2 strains may predominate, necessitating use of more toxic second line antiviral agents.[9] HSV-2 can disseminate in a minority of patients with AIDS leading to multi-organ involvement and severe morbidity.[10]

While CD4+ T-cell deficiency underlies this predisposition to high HSV-2 shedding rates and more severe disease, fundamental questions regarding more detailed mechanisms of immunosuppression remain unanswered. Though CD4+ T-cells mediate both humoral and cellular arms of the immune response, it remains controversial whether antibodies or cytolytic CD4+ and CD8+ T-cells are more important in control of HSV-2. Proper antigen processing and natural killer cell activity is likely to also be important in mediating HSV-2 containment, and both of these processes are abnormal in patients with AIDS.[11, 12] Animal models suggest that antibodies may provide early partial control of HSV-2 expansion while cell-mediated factors are responsible for late containment of the virus.[13, 14] An antibody-mediated vaccine did provide partial protection against HSV-1 acquisition.[15] Yet, HSV-2 is typically acquired before HIV-1 and an anti-HSV antibody response may already be established at the time of HIV acquisition. Moreover, vaccines that generate antibodies against key HSV-2 entry proteins have not yet provided protection against HSV-2 acquisition,[15, 16] and inherited agammaglobulinemia is not a risk factor for severe HSV-2 infections. Complicating this matter further is the fact that immune mechanisms that provide protection against acquisition may differ from those responsible for immune control during ongoing infection.

The most critical anatomic location for HSV-2 control has also yet to be identified. In animal models, rapid elimination of HSV-2 occurs due to tissue resident CD4+ and CD8+ T-cells that interface with lytically infected epithelial cells in skin and mucosa.[17, 18] In the human genital tract, tissue resident CD8+ T-cells reside at the dermal-epidermal junction at the precise site of HSV-2 release from neuron termini.[19–21] Analyses from human biopsies demonstrate that these cells exist in a persistent antiviral state and have a phenotype indicative of immunosurveillance.[22] CD4+ T-cells reside deeper in the dermis and, like CD8+ T-cells, accumulate in high numbers during severe reactivations and persist for months following viral clearance.[20] However, their function during clinical quiescence is unclear. Tissue resident T-cells also reside in sites of viral latency. In human trigeminal ganglia, CD8+ cells abut neuronal cell bodies with high copy numbers of HSV-1 DNA.[23] In mice, these cells utilize non-lytic mechanisms to limit reactivation.[24] The relative importance of HSV-2 containment in humans mediated in ganglia as opposed to mucosa is unknown.

Here we identify that HSV-2 shedding episodes are more frequent in HIV infected men and that extremely prolonged episodes occur more commonly in persons with AIDS. However, early viral expansion and late clearance rates, as well as peak episode viral loads, do not differ between HIV negative and positive men, regardless of CD4+ T-cell count. Next, we utilize mathematical models to demonstrate that release of HSV-2 from latency is likely to occur more frequently in HIV-1 seropositive persons regardless of CD4+ T-cell count while duration of cytolytic protection in genital mucosa is shorter in patients with CD4+ <200/μL. Free viral lifespan in mucosa also appears to increase with development of AIDS. These results imply that natural immunity at both latent and lytic sites of infection is important in limiting HSV-2 shedding rates and severity, and that prolonged shedding episodes in HIV infected persons may occur due to deficits in cell-mediated as well as humoral immune mechanisms within mucosal sites. We use our model simulations to infer that durable immunity in both latent and lytic sites are potential features of an effective therapeutic vaccine.

## Results

### Cohort characteristics

We matched 98 HIV-1 seropositive and 98 HIV-1 seronegative men according to country of origin, HSV-1 infection status and time since HSV-2 acquisition. Members of these cohorts have been described in other published studies. [25, 26] However, prior to this study, we did not perform detailed analysis of viral kinetics in these study participants. By design, demographic and clinical characteristics were similar amongst HIV+ and HIV- men. We further divided the HIV-1 positive cohort into three cohorts according to CD4+ T-cell count (<200, 200–499 and >500/μL). No participants were taking HSV-2 targeted antiviral therapy. HAART use was relatively evenly distributed amongst HIV men in different CD4+ T-cell strata. However, a higher percentage of men with AIDS (CD4+ T-cell <200/μL) were taking non-HAART antiretroviral therapy (p = 0.006), because HAART was not available when these studies were originally performed **(Table 1).**

**Table 1:** Clinical cohort characteristics.

Characteristic	HIV- (n = 98)	HIV+CD4 500+ (n = 34)	HIV+CD4 200–499 (n = 43)	HIV+CD4 <200 (n = 21)
**Median age (range)**	42 (25–76)	43 (29–56)	40 (29–64)	44 (27–66)
**HSV1-coinfected**	63 (64%)	19 (56%)	27 (63%)	17 (81%)
**Time since HSV2 acquisition**				
**1–9 years**	34 (35%)	11 (32%)	14 (33%)	9 (43%)
**10+ years**	26 (27%)	6 (18%)	14 (33%)	6 (29%)
**Unknown (no history at enrollment)**	30 (31%)	14 (41%)	10 (23%)	6 (29%)
**Unknown (history of lesions at enrollment)**	8 (8%)	3 (9%)	5 (12%)	0 (0%)
**Using ARVs**				
**None**	—	14 (41%)	18 (42%)	3 (14%)
**Non-HAART ARVs**	—	1 (3%)	5 (12%)	7 (33%)
**HAART**	—	18 (53%)	20 (47%)	10 (48%)
**Unknown**	—	1 (3%)	0 (0%)	1 (5%)
**Median HIV RNA (IQR)**	—	420 (15-10K)	3K (60-25K)	29K (5K-98K)
**HIV RNA category**				
**<10K**	—	26 (76%)	26 (60%)	10 (48%)
**10K-49K**	—	5 (15%)	10 (23%)	4 (19%)
**50K+**	—	3 (9%)	7 (16%)	7 (33%)
**Median CD4 count /μL (IQR)**	—	610 (535–735)	320 (240–400)	130 (85–160)
**Total episodes (a maximum of 10 episodes were counted per person)**	173	55	95	58

### Increased HSV-2 shedding rate, episode rate and episode duration in HIV+ infected men

**Fig 1:**
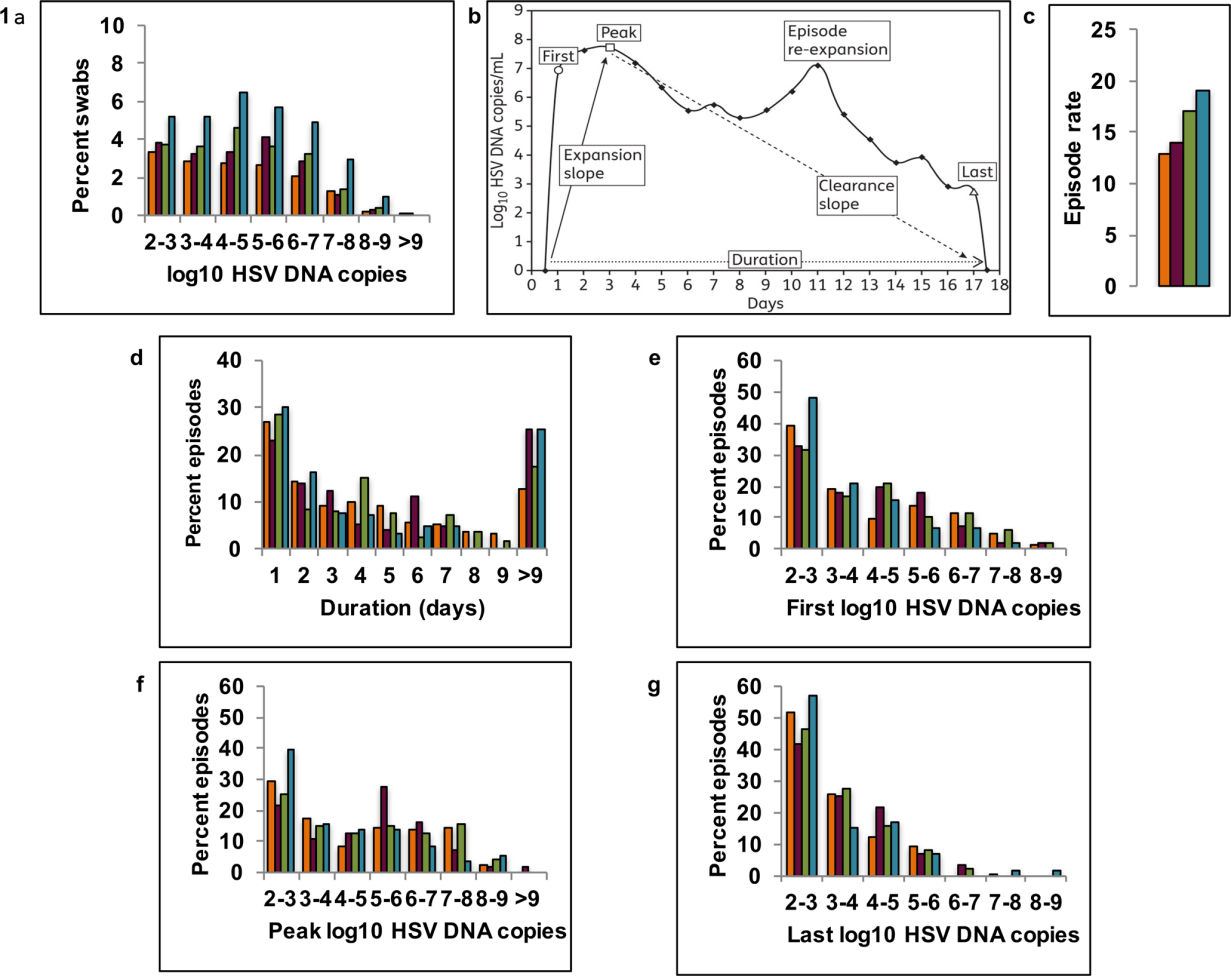
Increased HSV-2 shedding rate, episode rate and episode duration in HIV infected men. Frequency histograms of HSV-2 shedding in cohorts of HIV negative (orange), and HIV positive men with CD4+ T-cells >500/μL (magenta), 200-499/μL (green) and <200/μL (teal). (**A)** Quantitative shedding per swab; **(B)** Shedding episode classification scheme (adapted from reference 4; (**C)** Annualized episode rate; (**D)** Duration, (**E)** First positive viral load, (F**)** Peak positive viral load & (**G)** Last positive viral load per episode.

Study participants performed daily genital swabs for HSV-2 virus, which were analyzed using polymerse chain reaction (PCR). Cumulative HSV-2 shedding rate among HIV seropositive men (1375/6022 or 23% of swabs positive for HSV DNA) was higher than among HIV seronegative men (978/6448 or 15% of swabs positive for HSV DNA). For HIV positive men with CD4+ T-cell count >500/μL, HSV shedding rate was 19% (367/1960). For men with CD4+ T-cell count 200-499/μL, shedding rate was 21% (517/2495) and for those with CD4+ T-cell count <200/μL, shedding rate was 31% (491/1556). Frequency of shedding at both low and high viral loads was higher in HIV positive men and this effect was increasingly evident with decreasing CD4+ T-cell count **(Fig 1A)**.

Individual shedding rates were heterogeneous within each cohort. Median per person shedding rate was 11% (range: 0–68) in HIV negative men. For men with CD4+ T-cell count >500/uL, median per person shedding rate was 15% (range: 0–73). For men with CD4+ T-cell count 200–499 /uL, median per person shedding rate was 18% (range: 0–65). For men with CD4+ T-cell count <200/uL, median per person shedding rate was 25% (range: 2–82). Percentage of days with genital lesions was higher in men with lower CD4+ T-cell counts (8%, 5%, 11% and 15% for HIV negative and HIV positive with CD4+ T-cell count >500, 200–499 and <200/μL, respectively).

All shedding episodes were classified according to several fundamental characteristics **(Fig 1B).** Two features of episodes explain increased shedding rate in HIV infected men. First, annualized episode rate was higher in HIV infected men and increased as CD4+ T-cell count decreased below 500/μL **(Fig 1C, Table 2)**. Second, using an interval censoring approach to measure episode duration **(Methods)**, median duration was 1.8 days higher in HIV positive men with CD4+/μL <200 (p = 0.04) than in HIV negative men **(Table 2)**. A higher percentage of episodes exceeded 9 days in men with than without HIV-1 (regardless of CD4+ T-cell count) **(Fig 1D).** Summarized **(S1 Data)** and raw **(S2 Data** and **S3 Data)** are available in data files.

**Table 2:** Episode characteristics. Rate outcomes were compared by arm between persons using generalized estimating equations with a log link. Continuous outcomes were compared using generalized estimating models with an identity link. Significant differences are noted in bold and are limited to differences in episode rate and episode duration. All other episode characteristics are equivalent across cohorts.

Episode characteristics (n = 381)	HIV- (n = 173)	Test versus HIV-	HIV+CD4 >500 (n = 55)	HIV+CD4 200–500 (n = 95)	HIV+CD4 <200 (n = 58)
**Annualized rate of episodes per year (n = 572)**	13 (n = 275)	**Risk ratio (95% CI; p-value)**	1.0 (0.8, 1.4; 0.79)(n = 82)	1.3 (1.0, 1.7; 0.056)(n = 125)	**1.5 (1.1, 2.0; 0.018)(n = 90)**
**Starting HSV DNA copy**	4.1	**Average difference (95% CI; p-value)**	0.0 (-0.5, 0.5; 0.95)	0.2 (-0.2, 0.7; 0.31)	-0.4 (-1.0, 0.3; 0.26)
**Ending HSV DNA copy**	3.3	**Average difference (95% CI; p-value)**	0.2 (-0.2, 0.6; 0.18)	0.2 (-0.1, 0.5; 0.16)	0.3 (-0.2, 0.8; 0.26)
**Maximum copy (average)**	4.7	**Average difference (95% CI; p-value)**	0.2 (-0.4, 0.8; 0.48)	0.2 (-0.4, 0.7; 0.50)	-0.4 (-1.0, 0.2; 0.15)
**Upslope to maximum value (log 10 HSV DNA / day)**	4.3	**Average difference (95% CI; p-value)**	-0.6 (-2.4, 1.3; 0.56)	-0.5 (-2.2, 1.2; 0.53)	-1.1 (-3.0, 0.9; 0.28)
**Downslope from maximum value (log 10 HSV DNA / day)**	-1.8	**Average difference (95% CI; p-value)**	0.4 (-0.2, 1.0; 0.20)	-0.7 (-1.6, 0.1; 0.10)	-0.4 (-1.8, 0.9; 0.54)
**Duration using interval censoring (n = 547), days**	3.7(n = 260)	**Log rank (p-value)**	4.6 (0.11)(n = 78)	4.1 (0.38)(n = 123)	**5.5 (0.042)(n = 86)**
**Percent with lesions**	39 (23%)	**Risk ratio (95% CI; p-value)**	0.7 (0.3, 1.4; 0.28)	1.1 (0.7, 1.9; 0.70)	0.9 (0.4, 1.9; 0.78)
**Percent with no HSV rebound**	140 (81%)	**Risk ratio (95% CI; p-value)**	0.9 (0.8, 1.1; 0.45)	0.9 (0.8, 1.1; 0.32)	1.0 (-0.9, 1.1; 0.96)

### Equivalent viral expansion and clearance kinetics in HIV positive and negative men

All shedding episodes were also classified to capture early expansion and late clearance kinetics, as well as total viral production **(Fig 1B).** Median values for first, peak, and last HSV DNA copy number in shedding episodes did not differ statistically between HIV positive and negative men, or according to CD4+ T-cell strata **(Table 2)**. Frequency distributions of first **(Fig 1E),** peak **(Fig 1F)** and last (**Fig 1G)** HSV DNA copy number per episode appeared similar in all 4 cohorts. Early expansion and late clearance rates of HSV DNA in mucosa were, therefore, not associated closely with CD4+ T-cell count.

High first and peak HSV DNA values exceeding 10^6^ and even 10^7^ HSV DNA copies were common in all groups. Within each group, distribution of peak episode viral loads **(Fig 1F)** revealed only slightly higher values than first documented values **(Fig 1E)**, highlighting the rapid expansion of virus to peak levels within 24 hours in all cohorts. The distributions of first episode viral loads had generally higher values than the distributions of last episode values **(Fig 1G)**, indicating that early HSV-2 expansion is more rapid than late clearance, regardless of host immune status. Accordingly, median initiation to peak slope was considerably higher than peak to termination slope, though neither measure differed statistically amongst HIV negative and HIV positive cohorts **(Table 2).** Nineteen percent of episodes demonstrated viral rebound of at least 0.5 log following a period of clearance of at least 0.5 log: this did not differ statistically amongst the groups.

In summary, shedding episodes kinetics were strikingly similar in HIV negative and positive men, regardless of CD4+ T-cell count. HSV-2 shedding rate was higher in HIV positive men with low CD4+ T-cell counts because of a more frequent episode rate and slightly longer episode duration. On the other hand, episode expansion rate, clearance rate, re-expansion frequency and peak viral loads were equivalent among these populations.

### Mathematical model fit

We sought to identify a more detailed mechanistic underpinning for the increased shedding rate in HIV infected men by using mathematical model simulations. Our model consists of stochastic differential equations describing seeding of new HSV-2 lesions by virus released from ganglionic latency; infection of susceptible epithelial cells within a single ulcer with viruses from adjacent epithelial cells; seeding of new ulcers in spatially discrete regions by cell-free HSV-2 particles; linear clearance of cell free HSV-2; death of infected cells due to either viral lysis or T-cell mediated killing; and expansion of T-cells in the context of increasing burden of viral antigen. Model equations have been described elsewhere[27–29], and are detailed in the **Methods** and **S1 Fig**. Model parameters are described in the **Methods** and **Table 3.**

**Table 3:** Parameter ranges used for model fitting. Varied parameters were selected from uniform distributions. Fixed parameters were derived from prior model fitting or literature search **(Methods).**

Varied parameters	Units	Symbol	Low value	High value
**Neuronal release rate**	HSV DNA copies / day / genital tract	*ϕ*	25	250
**CD8+ T-cell decay rate**	log_10_ Days^-1^	δ	-4	-2
**Free-viral decay rate**	Days^-1^	*c*	2.5	14
**CD8+ T-cell local recognition**	log_10_ infected cells at which Θ is half maximal	*r*	1	3
**Maximal CD8+ T-cell expansion rate**	Days^-1^	Θ	0.6	5.0
**Fixed parameters**	**Units**	**Symbol**	**Value**	
**Cell-associated HSV infectivity**	DNA copy days/cell (viruses needed per day to infect one adjacent cell)	β_i_	5.4–8	
**Cell-free HSV infectivity**	DNA copy days/cell (viruses per day to initiate one ulcer)	β_e_	1e-11	
**Epidermal HSV replication rate**	log 10 HSV DNA copies/ cell / day	*p*	5	
**Viral production lag**	Days	ε	1.5	

It is important to note that the T-cell equation in the model is intended to capture the entirety of cell-mediated activity against HSV-2 infected cells. The model explicitly follows T-cells because the greatest amount of data exists to describe the expansion and clearance of these cells in genital mucosa. While we recognize that NK cell or antibody dependent complement mediated cytotoxicity may contribute in a meaningful way to clearance of infected cells,[30, 31] there is inadequate available dynamic and functional mucosal immune data during HSV-2 clearance to partition the cytolytic immune response into these more detailed components. Similarly, we do not explicitly differentiate between CD4+ and CD8+ T-cells because it is unknown what proportion of HSV-2 clearance can be attributed to each subset of cells in humans. Finally, the model is not able to distinguish a decrease in mucosal T-cell number from a decrease in T-cell function because whole genital tract T-cell counts cannot be measured in humans. However, our prior modeling has demonstrated that to maintain an equivalent shedding rate, a decrease in T-cell cytolytic function would need to be compensated by an overall increase in T-cell density.[27, 28] Therefore, our T-cell equation is intended to represent a summary measure of both number and function of all mucosal cytolytic cells with activity against HSV-2 infected cells.

We hypothesized that immunosuppression in HIV infected persons may reflect poor control within ganglionic as well as mucosal sites of infection, and via cell-mediated and / or humoral deficits. We therefore generated 1000 parameter sets in which the following values in the model were randomly selected from uniform distributions with realistic ranges: 1) rate of HSV-2 release from neural ganglia (a downstream measure of immune control within the ganglia), 2) duration of T-cell protection in genital mucosa, 3) expansion rate of T-cells in mucosa, 4) efficiency of cytolytic T-cell recognition of infected cells in the mucosa due to antigen presentation (measured by the number of infected cells at which expansion rate increases to its half maximal value) and 5) free viral lifespan in mucosa (**S1 Fig**). Ranges for parameter values are described in **Table 3**.

**Fig 2:**
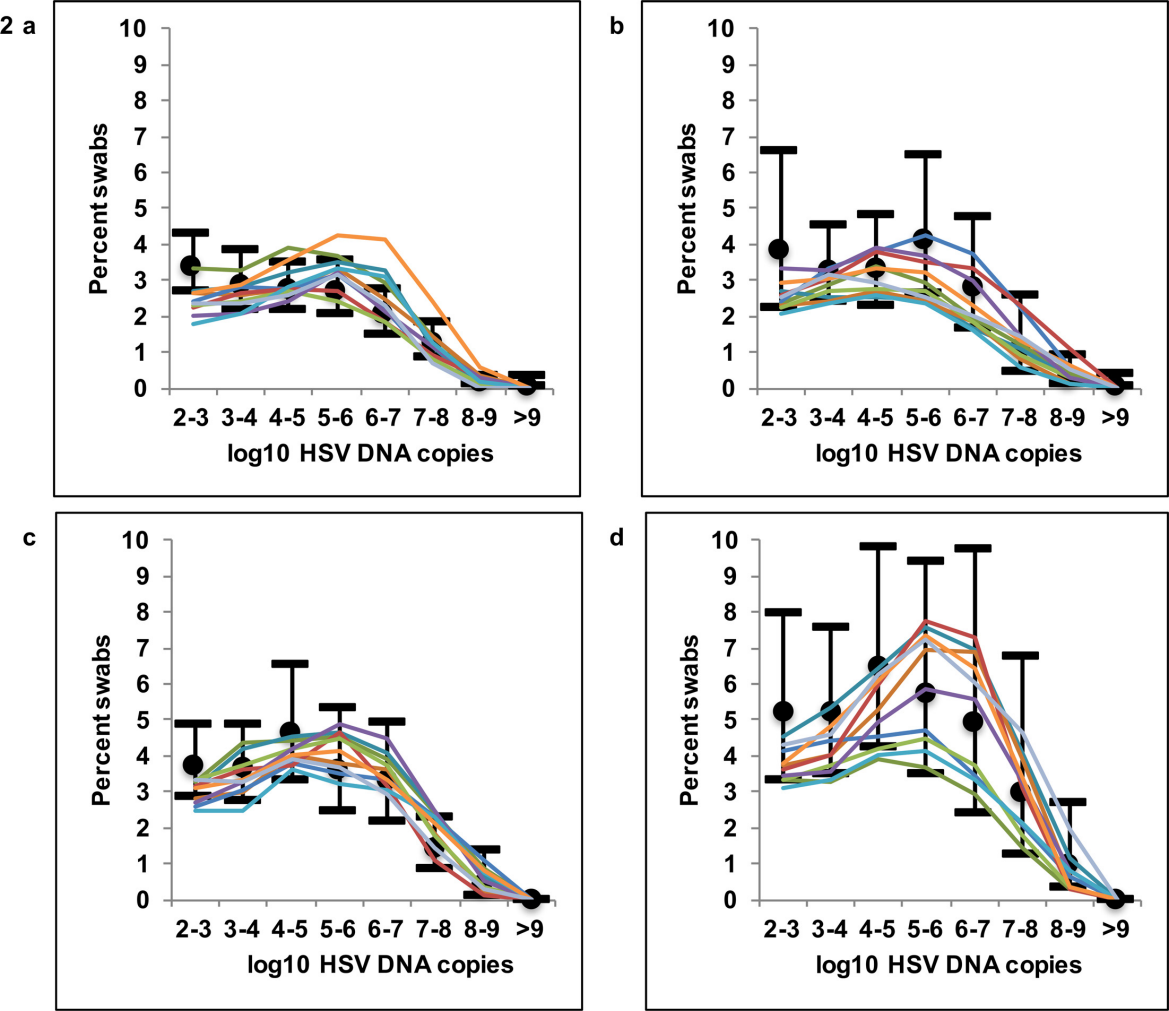
Mathematical model fit to quantitative shedding rate and episode rate. Ten mathematical model simulations of HSV-2 shedding (thin colored lines) in reference to empirical shedding data (median marked with black dot and 95% CI with black horizontal bars). Quantitative shedding per swab for **(A)** HIV negative, and HIV positive men with **(B)** CD4+ T-cells >500/μL, **(C)** 200-499/μL and **(D)** <200/μL.

Parameter sets were ranked according to fit to most kinetics features of the data described in **Fig 1**. The top 1% of best fitting parameter sets closely reproduced HSV-2 kinetic features for all cohorts. Model simulations recapitulated higher quantitative shedding rate as a function of decreasing CD4+ T-cell count **(Fig 2A–2D)**. Simulated HSV-2 episode rates fell within 95% confidence intervals for all cohorts and were generally lower in HIV negative versus CD4+<200/μL cohort men **(S2 Fig)**. Simulations with the top 1% of parameter sets for all 4 cohorts also accurately reproduced distributions of first **(S3 Fig)**, peak **(S4 Fig)** and last **(S5 Fig)** positive HSV DNA values during episodes.

We initially attempted to score simulations for accordance with episode duration histograms in **Fig 1D**. However, the model over fit to this episode characteristic resulting in poorer fit to the most clinically relevant data: quantitative shedding rate in **Fig 1A)**. We therefore described simulated episode duration relative to the actual data without formally scoring for fit. Only 67% of actual shedding episodes were of known duration in our cohorts, because the remaining 33% of episodes were ongoing either at the initiation or termination of the sampling period. As the longest episodes were the most likely to be of unknown duration, we classified episode duration with an interval censoring approach **(Fig 1D and S6 Fig)** in which the duration of these partially observed episodes was estimated based on existing data **(Methods)**. In all 4 cohorts, the model over-estimated percent of episodes of one-day duration as projected by interval censoring. Fit improved if only episodes of known duration were included for comparison to the model **(S6 Fig).**

#### Increased viral release rate from ganglia, decreased duration of T-cell protection and decreased clearance rate of cell free HSV-2 in HIV-infected men

**Fig 3:**
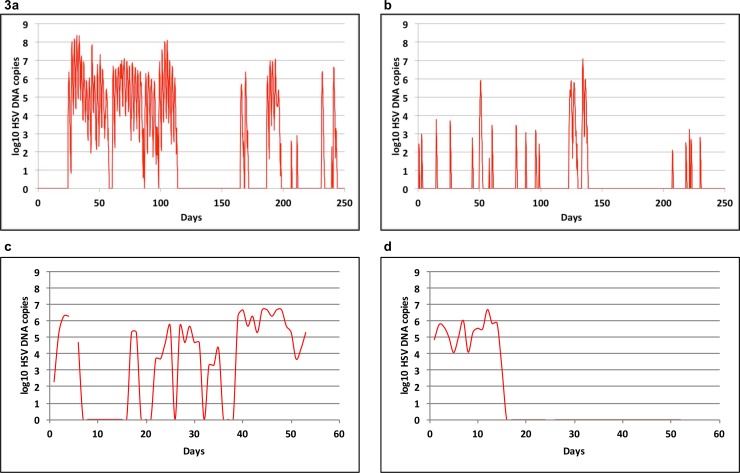
Increased HSV-2 shedding rate, episode rate and episode duration in HIV infected men. The red line indicates genital HSV-2 viral load over time. Model output for 300 days with parameters optimized for men with **(A)** HIV infection and CD4+ T-cell count<200/μL and **(B)** no HIV infection. Data from individual study participants with **(C)** HIV infection and CD4+ T-cell count<200/μL and **(D)** no HIV infection. Individuals in **(C)** and **(D)** performed daily swabs.

We used median parameter values for the top 5% of best fitting parameter sets in each of the 4 cohorts and observed characteristic, episodic HSV-2 shedding with prolonged simulations. When parameters optimized for men with CD4+<200/μL were utilized (**Fig 3A),** we noted a higher episode rate and longer episodes due to more frequent re-expansions than in simulations with parameters optimized for the HIV-1 uninfected cohort **(Fig 3B)**. The simulated episodes grossly resembled actual shedding patterns in HIV positive men with CD4+<200 (**Fig 3C)** and HIV negative men **(Fig 3D)**.

**Fig 4:**
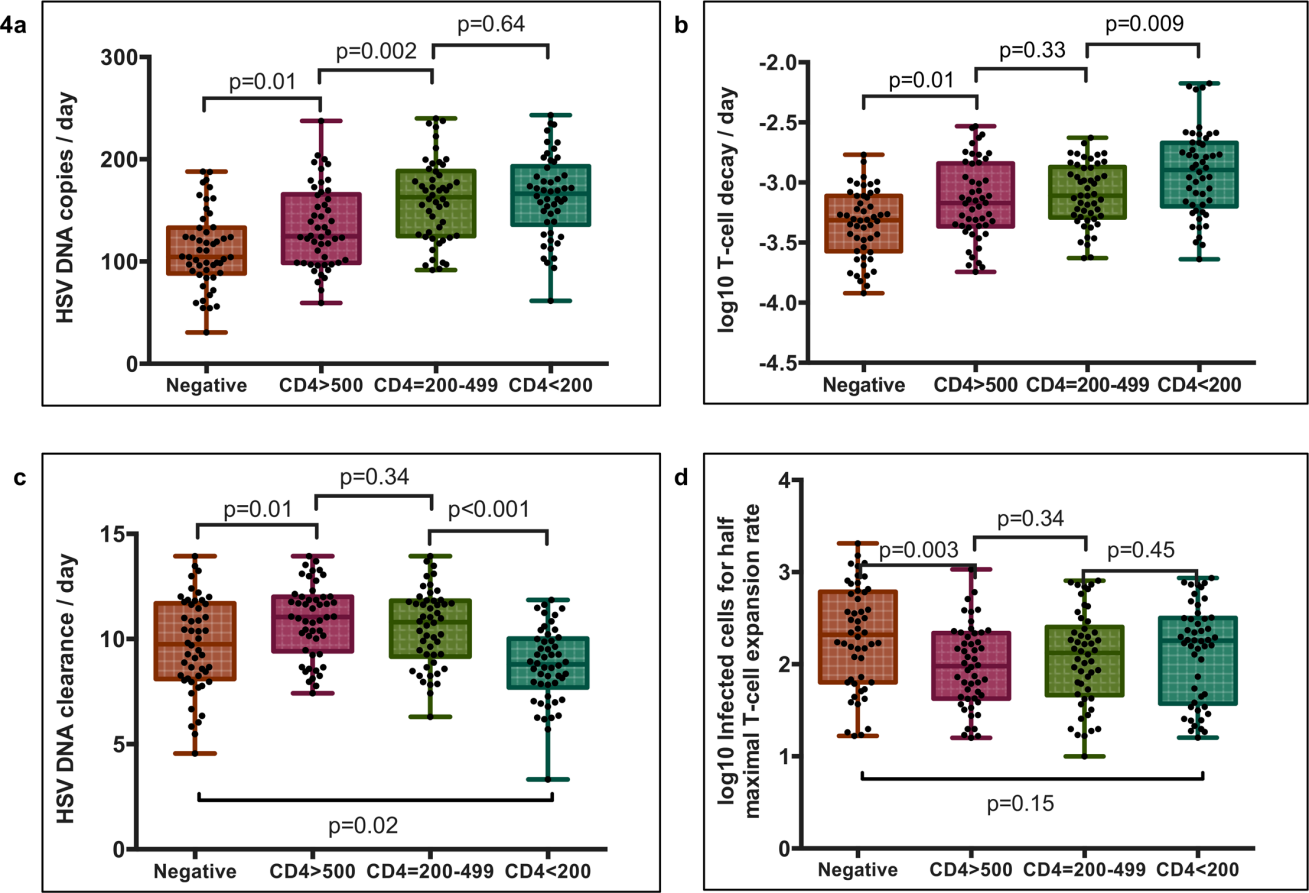
Immunologic determinants of increased HSV-2 shedding in HIV-1 infected men. Parameter sets represent the top 50 (5%) model fits for each group. Boxplots whiskers are inclusive of the full range of values. The box includes interquartile range and a median line. **(A)** HSV-2 release rate from ganglia to the genital tract, **(B)** Genital T-cell decay rate, **(C)** Genital HSV-2 DNA clearance rate and **(D)** Number of infected cells needed to stimulate half-maximal genital T-cell expansion. All comparisons are with Mann Whitney rank tests. Of note, genital T-cell levels in the model are intended as a surrogate measure of cytolytic immune potential.

To explain these observations, we compared values from parameter sets that accounted for the best 5% of fits across cohorts. We first noted considerable overlap amongst best-fit parameter values in all of the CD4+ T-cell count cohorts, suggesting that increased shedding in an individual is likely to be determined by the overlapping effects of multiple immunologic deficits. The rate of HSV-2 release from ganglia generally was higher in HIV infected persons with the largest interval difference between CD4+>500/μL and CD4+ 200-499/μL **(Fig 4A)**. Mucosal T-cell decay rate was also higher as a function of decrease in CD4+ T-cell strata amongst HIV infected men **(Fig 4B)**. We found a significant decrease in free viral clearance rate between the CD4+>200-499/μL and CD4+<200/μL groups, suggesting a loss of humoral immune function **(Fig 4C)**. An increase in clearance rate of HSV-2 was noted between HIV negative men and HIV positive men with CD4+>500/μL. Finally, the number of HSV-2 infected cells required for a cytolytic T cell response to infection was estimated to be lower in men with HIV and CD4+ T-cell count>500/μL versus HIV negative men **(Fig 4D):** this may suggest impaired antigen presentation in patients with HIV. Cytolytic T-cell expansion rate was equivalent in all 4 cohorts (not shown).

**Fig 5:**
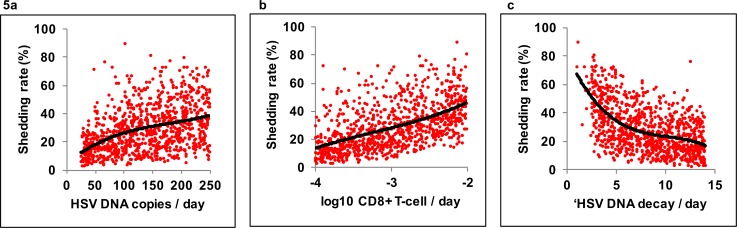
Individual determinants of increased HSV-2 shedding rate. Critical model predictors of shedding rate in 10-year simulations with 1000 parameter sets. Curves in all panels indicate best fit with a polynomial model while dots are single simulations. **(A)** HSV-2 release rate from ganglia to the genital tract (R^2^ = 0.17), **(B)** Genital T-cell decay rate (R^2^ = 0.29), **(C)** HSV-2 DNA clearance rate (R^2^ = 0.30), predict shedding rate to various degrees. Genital T-cell levels in the model are intended as a surrogate measure of cytolytic immune potential.

To further delineate which parameters were most likely to vary as a function of CD4+ depletion, we estimated the impact of parameter variability on overall shedding rate in all 1000 simulations. We noted a wide possibility of shedding rates at each parameter value **(Fig 5A–5C)** again highlighting that no single immune parameter predicts a person’s shedding rate. The three parameters that differed most across CD4+ T-cell strata **(Fig 4)**, including free viral release rate **(Fig 5A)**, T-cell decay rate **(Fig 5B)**, and free viral clearance rate **(Fig 5C)** had a large impact on shedding rate. However, the remaining two parameters, number of cells required for a T-cell response to infection and T-cell expansion rate, were less important correlates of shedding rate (R^2^ = 0.07 and R^2^ = 0.05 respectively). We conclude that these two parameters are less likely to explain major differences in shedding rate according to HIV infection or CD4+ T-cell count.

We next interrogated the more specific effects of these parameters on HSV-2 shedding in these model simulations. These simulations assume daily sampling in keeping with the clinical sampling protocol. We identified that 1) free viral release rate correlated with increased episode rate (R^2^ = 0.35), 2) T-cell decay rate correlated slightly with average episode duration (R^2^ = 0.07), and 3) free viral clearance rate correlated inversely with average episode duration (R^2^ = 0.07) as well as episode rate (R^2^ = 0.34). Of note, the decrease in episode rate due to higher free viral clearance is only held under the assumption of daily sampling because higher viral clearance rates lead to a higher frequency of brief episodes lasting <24 hours. No other highly positive correlations were noted between these parameters and other episode kinetic features including peak viral load, first positive viral load during an episode or last positive viral load during an episode (not shown).

### An immunodeficiency cascade in HIV-1 infected persons

We next performed prolonged simulations using median values from the top 5% of parameter sets in both HIV-negative men and men with CD4+ <200/μL. These analyses were performed with the assumption of continuous rather than daily sampling to characterize the role of brief asymptomatic shedding episodes, which are common in both HIV-infected and uninfected persons with genital HSV-2 infection, but often missed with daily sampling protocols.[3, 25] Simulations continued for ~15 years until there were 500 episodes generated in each group: shedding rates were 14% and 27.8% respectively, with the HIV negative and CD4+<200/ μL parameter sets.

**Fig 6:**
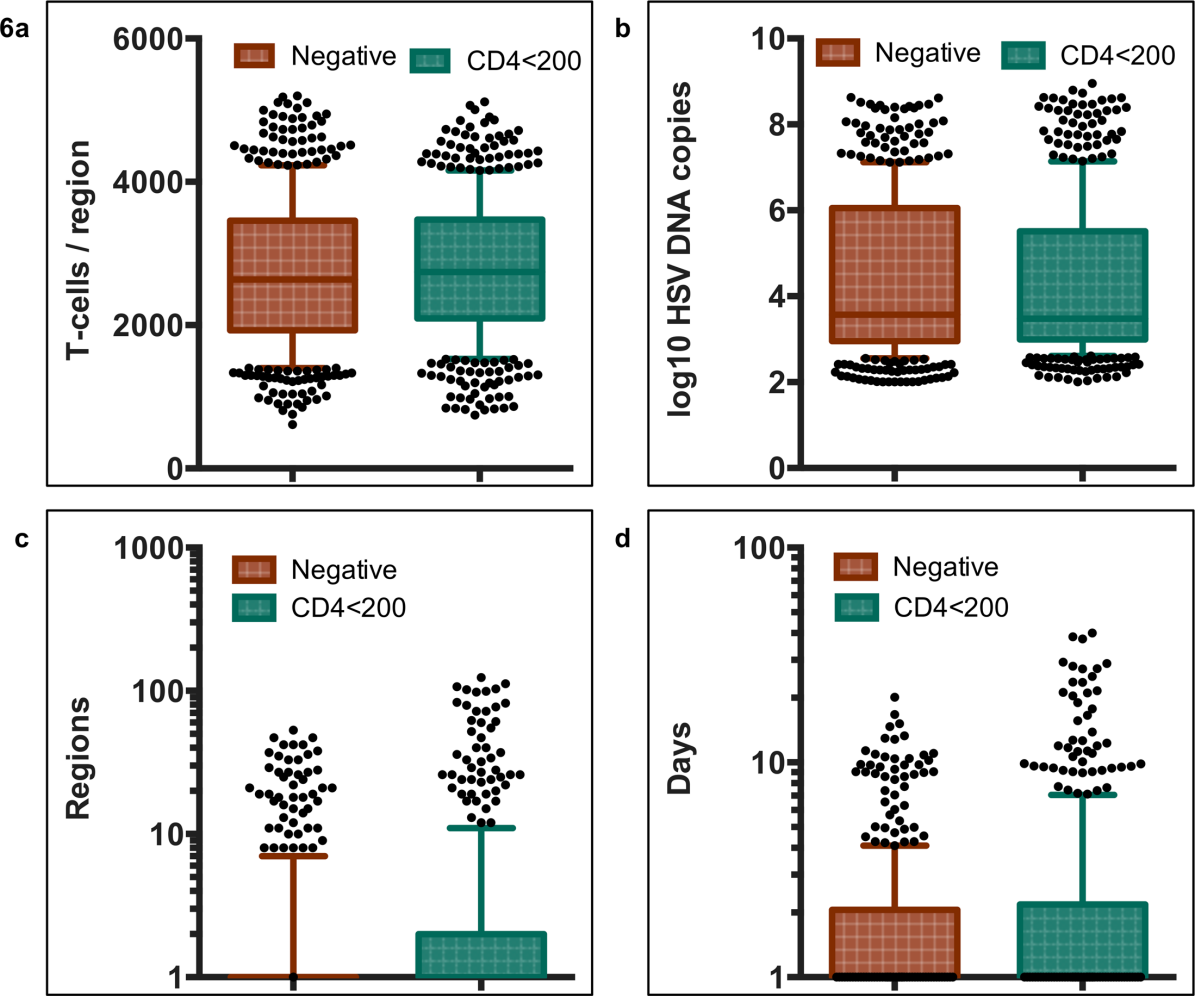
HSV-2 shedding episode characteristics in HIV-1 negative and positive men with CD4+ T-cells count <200/μL. 500 simulated episodes with parameters optimized for HIV negative (orange) and HIV positive men with CD4+ T-cells <200/μL (teal). Boxplots whiskers are inclusive of 90% of values and dots are simulations outside of this range. The box includes interquartile range and a median line. **(A)** Genital T-cell density in model region of episode initiation, **(B)** Peak HSV DNA copy number, **(C)** Number of infected regions per shedding episode, **(D)** Episode duration. Comparisons for each of the episode characteristics were not statistically significant with non-parametric Mann Whitney rank tests. However, simulations with CD4+ T-cells <200/μL had higher proportions of episodes involving more than 50 regions **(C)** and lasting more than 10 and 20 days **(D)**.

We identified immunodeficiency at each step in the HSV-2 pathogenesis cycle in men with HIV-1 and CD4<200/uL. HSV-2 mucosal seeding from ganglia occurred more frequently leading to an increased annualized episode rate (41 versus 31 in HIV negative men). The range of cytolytic T-cell densities (a surrogate of overall cytolytic potential against HSV-2 infected cells in our model) at episode onset in micro-regions of mucosal reactivation, was predicted to be equivalent in the HIV negative and CD4+ T-cell<200/μL cohorts **(Fig 6A).** Owing to corresponding T-cell densities at episode initiation and in concordance with empirical data **(Fig 1F)**, the distribution of simulated peak viral loads did not differ between groups **(Fig 6B)**. We observed profound spatial heterogeneity of T-cell density across micro-regions in both cohorts **(S1 and S2 Movies)** Episode initiations in low T-cell density anatomic sites allowed for higher viral loads, and more prolonged episodes, regardless of CD4+ T-cell cohort.

Equivalent mucosal cytolytic T-cell level distributions between men without HIV infection and with CD4+<200/μL, does not imply equivalent cytolytic immune function in the mucosa. Despite two-fold higher shedding rates, cytolytic T-cell densities were no higher in in the CD4+ T-cell <200/μL cohort, primarily because T-cell decay rates were approximately twice as high in this group **(Fig 4B)**. Therefore, the model predicts that turnover of mucosal cytolytic immune cells occurs much more frequently in men with AIDS.

The model predicted that prolonged episode duration occurred not because of slower clearance of infected cells within single micro-regions, but rather due to slower clearance of free virus **(Fig 4C)**. This promoted a higher percentage of episodes with substantial secondary seeding of HSV-2 into dozens of spatially discrete mucosal locations, allowing wider spatial spread of HSV-2 during approximately 10% of episodes **(Fig 6C)**. Though rank distribution of episode duration was not statistically different between the groups, a higher percent of episodes exceeding 10 days (6% vs 2.8%, p = 0.01) and 20 days (2.8% vs 0.2%, p<0.001) were noted in the simulations of the group with CD4+ T cell <200/μL **(Fig 6D).** These exceptionally long episodes were notable for multiple HSV-2 re-expansion phases and wide spatial dissemination of virus **(S2 Movie),** and correspond to severe persistent lesions in patients with AIDS.

## Discussion

A formidable challenge in understanding the immune response to viral infections is to synthesize complex and sometimes conflicting data pertaining to different arms of the immune response in various anatomic compartments into a more comprehensive picture of pathogenesis at the level of the whole person. Whereas associations can be drawn between cross sectional measures of an antibody or T-cell response against a virus and relevant clinical outcomes, it is considerably more difficult to demonstrate a causal link.[32] Human data is typically observational and critical host and pathogen variables cannot be regulated. Highly controlled animal models allow better mechanistic understanding, but often do not accurately recapitulate all critical features of human infection. For instance, in murine models of HSV-2 infection, both cellular and humoral immune mechanisms are capable of independently containing or preventing viral infection, and this control can be demonstrated in latent or lytic infected tissue.[13, 14, 17, 18, 24] Yet, vaccines that are fully protective in animals are ineffective in humans, and no mechanistic correlate of protection has been identified for either HSV-2 acquisition or reactivation.

We used a mathematical modeling approach to develop hypotheses regarding which components of the HSV-2 targeted immune response degenerate during progressive HIV infection. Our modeling suggests that HSV-2 is released from neural ganglia at a higher rate in patients with AIDS, leading to a higher frequency of shedding episodes. Given that HSV-specific T-cells can limit ganglionic reactivation in murine models, this prediction may imply that cell-mediated immunity within neural tissue is a determinant of a patient’s natural history though human data is needed to test the true mechanisms of anti-HSV-2 immunity within neurons.

In persons with CD4+<200/μL, a small subset of shedding episodes is extremely prolonged. Our model suggests that this may be due to seeding of high number of adjacent regions during these episodes. The probability of secondary seeding is enhanced by slower clearance of free HSV-2 particles during late HIV infection, possibly suggesting a defect in the HSV-2 specific antibody response. Surprisingly, the free viral clearance rate is predicted to increase in HIV infected men with CD4+ >500/ μL relative to men without HIV (**Fig 4C**). We lack an explanation for this model prediction, though it is possible that the paraproteinemia associated with early HIV infection boosts free viral clearance.[33]

We previously demonstrated that episode prolongation beyond 2–3 days is due to viral re-expansion rather than prolonged sustained shedding at a constant viral load.[29] Our model predicts that this re-expansion is due to free viral seeding of new regions. The finding in the current study that viral re-expansion is no more common in men with HIV infection likely reflects the fact that single day episodes are equally probable in each group, and episodes beyond 3–4 days represent a minority of episodes in all cohorts.

An unexpected finding was that the immediate potency of HSV-2 immune responses within single infection microenvironments appears similar in HIV positive and negative men regardless of CD4+ T-cell count. This observation holds during early, peak and late stages of a reactivation as evidenced by equivalent distributions of viral loads at each of these time points in all HIV negative and positive cohorts. Our model predicts an equal distribution of cytolytic T-cell effect at episode onset regardless of CD4+ T-cell count. This result is consistent with histopathologic studies that reveal infiltrates of HSV-2 specific CD4+ and CD8+ T-cells, which may represent sites of prior HSV-2 containment.[34] Our model predicts that the lifespan of tissue resident cytolytic T-cells is shorter in HIV-1 infected men, allowing for T-cell densities equivalent to those in HIV-1 seronegative men, despite a more frequent need for local reconstitution. Tissue resident, HSV-2 specific T-cell turnover and replacement is therefore predicted to become increasingly dynamic as CD4+ T-cell decreases during HIV-1 progression. An alternative possibility is that T-cells are less effective killers in patients with low CD4+ T-cell count, though this would likely imply the need for higher density of HSV-specific T-cells in genital mucosa to explain observed shedding patterns. Overall, these results suggest that dynamic deficits in tissue resident T-cells or other cytolytic mediators in genital mucosa, as well as less effective mucosal antibodies, may play critical roles in limiting control of HSV-2.

Conversely, model simulations, with decreased rate of viral release from ganglia, increased tissue resident lifespan of mucosal cytolytic T-cells, and high free viral clearance rates, resulted in lower shedding rates. The implications for promising immunotherapies are significant. Classical attempts to establish immunologic correlates of protection have relied on sampling of blood following vaccinations. However, tissue resident T-cells are fundamentally involved in mediating protection and are not homogeneously dispersed in blood. Here, we demonstrate that tissue resident immunity in tissues with latent and lytic viral infection may play important, but quite different roles in limiting viral shedding. Tighter virologic control in ganglia is predicted to limit shedding episode frequency while enhanced protection provided by genital tissue T-cells limits the probability of viral re-expansion and therefore prevents extremely prolonged episodes.

Several limitations of our study are important to note. Our model’s description of mucosal immunity reflects the available experimental data and does not capture the extraordinary complexity of a coordinated protective mucosal response. While HIV-1 infection specifically leads to CD4+ T-cell depletion, we do not model CD4+ T-cells separately from CD8+ T-cells. Although CD8+ T-cells can eliminate infected cells via contact mediated lysis, and CD4+ T-cells contain infection by secreting IFN-gamma in mice,[14] it is unknown whether these cells exert independent, additive, or synergistic control of HSV-2. Tissue resident CD8+ T-cells are likely to be divided into distinct subsets. For instance, the roles of antigen specific versus bystander tissue resident CD8+ T-cells differ and are also not separated in our model.[35] Natural killer cells may be responsible for clearance of a percentage of infected cells early after a reactivation. For these reasons, the T-cell variable in our model represents a surrogate for the overall intensity of regional HSV-specific cytolytic activity in the genital mucosa, which is in turn likely to be determined by T-cell density, functionality, breadth, and subset synergy.

Similarly, our description of immune pressure in the ganglia is reduced to a single model parameter: rate of HSV-2 release from neuron endings into genital mucosa. While we have demonstrated that local levels of tissue resident T-cell in genital mucosa expand in the setting of replicating and spreading HSV-2, and slowly contract between HSV-2 shedding episodes,[5, 19–21, 36] it is unknown whether similar dynamics apply in the ganglia, or whether T-cell concentrations are more stable over time and across spatial gradients. We therefore do not implicitly model the impact of T-cells or other arms of immunity within ganglia.

Our model overestimates the proportion of one-day episodes in all cohorts. A possible explanation is that detectable virus duration is slightly misclassified in model simulations. Computationally, we assume that cell-free virus is immediately captured with swabbing. In reality, the first infected cell is separated from the skin surface by a layer of 10–20 cells. If the delay between replication in a first infected cell and detection of HSV-2 at the epidermal surface is substantial, many rapidly cleared episodes that would be missed with daily sampling protocols in study participants are observed as one-day episodes in model simulations. Indeed, past studies that employed sampling every 6 hours in persons with and without HIV demonstrated that most detected episodes are shorter than 12 hours in duration,[3, 25] which is in accordance with our model simulations.

Finally, while our model establishes a general framework for poorer containment of HSV-2 shedding in HIV infected persons, the etiology of enhanced shedding in an individual patient is likely to be highly variable and may reflect more significant deficits in specific components of the immune response than others. Our categorization of men within different CD4+ strata only provides a partial classification of their antiviral immune status. Despite statistically significant differences between best-fit parameter values in the model, there was significant overlap of values amongst the CD4+ strata, and single parameter values did not reliably predict shedding rate. Even with stable parameters of viral replication and immune response, we have shown that shedding patterns in an individual are highly unpredictable over short time frames.[37] Given the multifactorial and dynamic determinants of a robust or impaired anti-HSV-2 response across patients, we surmise that there is unlikely to be a single mechanistic correlate of risk or protection against established HSV-2 infection.

In summary, we identify that increased HSV-2 shedding in HIV infected persons is a result of increased episode rate, as well as prolonged episode duration. These two factors are predicted to relate to a combination of shortened lifespan of mucosal T-cells, impaired mucosal clearance of free virus, and general immune deficits within ganglia. Immunotherapies that augment immune responses in both latent and lytic tissue sites of HSV-2 infection may be important in limiting infection.

## Materials and Methods

### Study subjects

All research was approved by the University of Washington Institutional Review Board (IRB) and all clinical investigation was conducted according to the principles expressed in the Declaration of Helsinki. Written informed consent was obtained from the participants. Data for this study were selected from the database of viral shedding studies performed between 1993 and 2008 in the University of Washington Virology Research Clinic, Seattle, WA.[25, 26] We studied HIV-1 positive and matched negative HSV-2 infected men who performed daily genital swabbing for detection of HSV-2 by polymerase chain reaction (PCR). The HIV-1 negative men were matched on country of origin, HSV-1 infection status and time since HSV-2 acquisition in categories of 1–9 years, 10+ years, and unknown (with or without a history of lesions). All study participants were off HSV-2 therapy. We included all HIV-1 seropositive men who contributed at least 30 consecutive days of genital swabbing and diaries and for whom there were available controls also with sufficient swabbing from the same country and the same gender. The techniques for collection and transport of swabs were published previously.[4–6] Each participant contributed approximately 60 days of swabs to the analysis. We did not include women in the analysis because we lacked sufficient numbers of HIV-1 infected women to form a comparator cohort among women.

### Laboratory methods

HSV serologic testing was performed by Western blot.[38] Swabs were placed into vials containing 1 mL of PCR transport medium and refrigerated until laboratory processing. HSV DNA was detected using a quantitative PCR assay, and the HSV DNA level was expressed as copies per mL of transport medium with the cutoff of 150 copies per mL for defining positive samples.[39] Laboratory personnel were blinded to clinical data.

### Viral Shedding outcomes

Shedding rate was defined as number of swabs with HSV DNA detected by PCR out of swabs collected. Lesion rate was computed as number of days with reported lesions divided by the number of days with swabs collected. Shedding episodes were defined by a series of consecutive swabs containing HSV DNA ≥ 150 copies/ml, and not including more than one consecutive time point with a negative or missed swab. Episodes of certain duration were those that started and ended with two negative swabs. To ensure that results were not subject to undue bias based on overrepresentation of study subjects who contributed more data, we described the first ten episodes observed from each subject. We described each shedding episode according to its duration, peak copy number, expansion rate and decline rate **(S1 Fig)**, and arranged all outcomes in frequency histograms to define ranges for these outcomes.

### Shedding episode frequency

To calculate relevant population-level estimates of annualized episode rate, we enumerated the number of shedding episodes initiated during the observation period, divided this number by the total number of daily swabs performed and multiplied by 365. We calculated episode frequency with all available swabs.

### Shedding episode duration

As swabs were taken every 24 hours, episodes may have initiated within 0–24 hours of the first positive swab of the episode, and terminated within 0–24 hours of the last positive swab. We assumed that the midpoint of this interval (12 hours) would provide an unbiased estimate of length. To account for the fact that duration of some episodes could not be observed when shedding was present on the first or last swab of the session, we constructed an additional definition of episode that allowed the duration to be uncertain. The time to completion of an episode was then a survival outcome, which could terminate as a censored episode; we performed interval censoring to estimate duration of these episodes.[40] This technique uses the assumption of independent censoring to estimate that censored episodes last, on average, as long as other episodes that were observed to persist for an equivalent or greater duration. Because some episodes using the first definition were separated from adjacent episodes only by missed swabs, the second method of computing duration can aggregate episodes defined by the first method and can result in fewer observed episodes overall.

### Shedding episode peak, expansion and decay

For measures of episode peak copy number, expansion and decay, we only included episodes of known duration since we were not otherwise certain to capture either the start or termination. We defined episode peak as the maximum copy number obtained over the episode. We calculated the rate of viral increase from initiation to the estimated peak of each episode by computing the slope of a linear regression line over the copy numbers up to and including estimated maximum copy; for this calculation, we set the time of the most proximal negative swab at 0.5 days prior to first positive. We calculated the rate of decrease from peak to termination of each episode in a similar fashion by setting the time of termination to 0.5 days post the last positive. We calculated a median for each of these two slope measures based on results from all included episodes. We identified proportion of episodes with and without re-expansion (defined as a rise in HSV copy number by 0.5 logs after a prior decline within the episode of at least 0.5 logs): of note, this measure underestimates the true frequency of viral re-expansion as studies with more frequent sampling (every 2 and 6 hours) identified that viral rebound is a nearly universal feature of episodes lasting more than 3 days.[27, 28]

### Statistical methods for comparisons

Rate outcomes like shedding and lesion rates were compared by arm between persons using generalized estimating equations models with a log link, to estimate risk ratios for shedding rates, lesion rates, and episode rates by group-level covariates like HIV status and CD4 count. Continuous outcomes like maximum copy obtained per episode were compared using generalized estimating models with an identity link.

### Mathematical model simulations

The *in silico* simulation model in this paper consists of 200 hexagonal micro-regions of infection in the genital mucosa.[27–29] These regions are linked by the ability of: 1) neurons to randomly release virus into any of the 200 regions and initiate shedding episodes by infecting a single keratinocyte in these regions, and 2) cell-free virus from one region to seed an adjacent hexagonal region leading to multiple concurrent foci of infection. Past versions of the model assumed immunologic overlap between model regions. However, we identified that this further complexity is not needed in the model to ensure fit to the data. The 200 micro-regions are arrayed in a two-dimensional matrix such that cell-free HSV from a region can only infect a maximum of six other regions **(S2 Fig)**. For edge regions, there are only 4 contiguous regions at risk.

The HSV-2 replication cycle and immunologic response to infected cells can occur concurrently in multiple hexagonal regions and is described mathematically in **S2 Fig.** Equations are outlined below:

ΔS(i=1…200)=[λ−(βi×Si×Vi)−(βi×Si×Vneu)−(βe×Si×Ve)]Δt

ΔI(i=1…200)=[(βi×Si×Vi)+(βi×Si×Vneu)+(βe×Si×Veadj)−(a×I)−(f×I×E)]Δt

ΔE(i=1…200)=[(Fi×θ×E)−(δ×E)]Δt

ΔVneu(i=1…200)=[ϕ−(βi×Si×Vi)−(c×Vneu)]Δt

ΔVi(i=1…200)=[(p*I)−(a×Vi)−(βi×Si×Vi)]Δt

ΔVe(i=1…200)=[(a×Vi)−(c×Ve)]Δt

λ=d÷(S−S0)

Fi=I/(I+r)

Veadj=Ve_from_6_adjacent_regions

Vetot=Ve1+Ve2+…+Ve200

Vitot=Vi1+Vi2+…+Vi200

In the model, HSV-2 (V_neu_) is randomly released from neuronal endings into the dermal-epidermal junction at a certain rate (*ϕ*). Neuronal HSV-2 is cleared from the genital tract at rate (*c*) during which time it can infect local epithelial cells (S). If an infection occurs, then one of the 200 genital tract regions is randomly selected as the site of infection take off. Viral infectivity (β_i_) determines how effectively cells are infected by HSV. If an epithelial cell becomes infected (I), then it dies via direct lysis at rate (a) or via CD8+ lymphocyte (E) mediated lysis at a rate (*E* × *f*). *p* is the rate of viruses produced by an infected cell per day. Re-growth of susceptible keratinocytes in a single region occurs according to a growth rate, λ or *d*÷(*S*0−*S*) with growth limited by S0, the carrying capacity of the system. The formation of a genital lesion is accompanied by accumulation of CD8+ lymphocytes at peak rate (θ), followed by slow decay of these cells at rate (δ). θ/2 occurs when infected cells are equal in number to parameter *r*, which represents how many epithelial cells need to be infected prior to half-maximal CD8+ expansion. Cell-associated HSV-2 (V_i_) is differentiated from cell-free HSV (V_e_) in the model: cell-associated particles can passage from cell-to-cell within one ulcer micro-environment as soon as they form within a cell and convert to cell-free virus (V_e_) after infected cells rupture. V_e_ can initiate formation of new plaques within adjacent regions by local seeding in our model, and are assigned an infectivity parameter (β_e_). Parameter (ε) is included to account for delay in viral production within secondary plaques after seeding. We use V_etot_ which is the sum of all cell-free virus in the 200 regions as the variable to assess congruence with our empirical shedding data in HIV infected and uninfected persons as cell-free virus is detected on our genital swabbing protocols.

We calculated the reproductive number *R* = (*p*×*βi*×*S*0)÷((*a*+(*f*×*E*)×*c*) continually during each simulation. The reproductive number is the average number of cells that an infected cell would infect assuming the presence of CD8+ T-cells. For our spatial models, which divided the genital tract into 200 separate regions that were susceptible to viral replication, we calculated R separately within each region.

All simulations were performed using C++. The model was solved stochastically due to the random nature of shedding episode initiation and clearance, and to account for frequent presence of low numbers of infected cells: at each time step, integer values for equation terms were drawn randomly from binomial distributions. Model variables were updated at a narrow time interval (0.001 days). Graphical output was made with Prism and Microsoft Excel.

### Model parameter search

One thousand unique parameter sets were generated by randomly selecting the following parameter values (*ϕ*, c, θ, *r* and δ) using Latin hypercube sampling from square distribution functions of each parameter. The distribution functions were constructed by creating wide ranges around best-fit values from model fitting in previous simulations of the model. Only model parameters that might vary in the context of suppressed CD4+ T-cell function were varied in the analysis: these parameters include neuronal release rate of HSV-2 (*ϕ*), peak CD8+ expansion rate (θ), infectious burden required for CD8+ expansion (*r*), CD8+ decay rate (δ), and free viral clearance rate (*c*). All other parameters were fixed based on prior model fitting and literature review, [5, 27–29, 36] and are described in **Table 3.**

Simulations were performed over 10-years. We noted that each parameter set required a 6-month equilibration period for establishment of a relatively stable viral shedding rate, and total density of CD8+ T-cells. We therefore started recording data only after allowing a one-year model run. Simulation data was recorded with daily sampling as in the study protocol, as well as with continuous sampling to estimate the percentage of rapidly cleared episodes.

### Assessing mathematical model equivalence with empirical data

We allowed an unbiased assessment of our multidimensional parameter space by testing whether different randomly selected parameter sets faithfully reproduced our empirical data. Accordingly, we did not solve the model by performing incremental discretization techniques. Instead, model simulations with each unique parameter sets were assessed for congruence with cumulative measure of core shedding summary measures (1. quantitative shedding rate, 2. episode rate, 3. first positive copy number of episodes, 4. last positive copy number of episodes, and 5. peak positive copy number of episodes), which contained a total of 31 data bins. Consistency of model output with episode duration was assessed graphically but not formally scored. We assembled the modeled data exactly as it was gathered in the clinical protocols by sampling every 24 hours.

Each unique parameter set was assigned a residual sum of squares score by weighting each of the 5 summary measures equally, calculating least residual sum squares between model simulations and the closest confidence interval bound of the actual data for each of the 31 data bins and summing these measures. Model values that fell within 95% confidence intervals for bin values were given a least squares score of zero. The top 5% (or 50) best-fit parameter sets for fit to shedding in the HIV infected and uninfected cohorts were selected for comparison. Parameter values in these sets were compared using non-parametric analyses. To develop emergent properties of the model for the different HIV cohorts, we selected median parameter values from the top 50 parameter sets for each of the 4 clinical cohorts, and performed prolonged model realizations.

## Supporting Information

S1 Data This file includes histogram data for Figs 1 and 2. (XLSX)

S2 Data This file includes raw shedding data. (XLSX)

S3 Data This file include labels for S2 Data. (DOC)

S1 Fig Mathematical model (adapted from reference). **(A)** Micro-regions are linked virally because cell-free HSV-2 can seed surrounding regions. **(B)** Schematic for HSV-2 infection within a single genital tract microenvironment.
(PDF)

S2 Fig Mathematical model fit to HSV-2 episode rate. Ten mathematical model simulations of HSV-2 shedding (colored dots) in reference to empirical shedding data (median marked with black dot and 95% CI with black horizontal bars). Annualized episode rate for **(A)** HIV negative, and HIV positive men with **(B)** CD4+ T-cells >500/μL, **(C)** 200-499/μL and **(D)** <200/μL.
(PDF)

S3 Fig Mathematical model fit to early HSV-2 expansion. Ten mathematical model simulations (thin colored lines) in reference to empirical shedding data (median marked with black dot and 95% CI with black horizontal bars). First positive HSV DNA copy number per episode for **(A)** HIV negative, and HIV positive men with **(B)** CD4+ T-cells >500/μL, **(C)** 200-499/μL and **(D)** <200/μL.
(PDF)

S4 Fig Mathematical model fit to peak episode viral load. Ten mathematical model simulations (thin colored lines) in reference to empirical shedding data (median marked with black dot and 95% CI with black horizontal bars). Peak positive HSV DNA copy number per episode for **(A)** HIV negative, and HIV positive men with **(B)** CD4+ T-cells >500/μL, **(C)** 200-499/μL and **(D)** <200/μL.
(PDF)

S5 Fig Mathematical model fit to late HSV-2 clearance. Ten mathematical model simulations (thin colored lines) in reference to empirical shedding data (median marked with black dot and 95% CI with black horizontal bars). Last positive HSV DNA copy number per episode for **(A)** HIV negative, and HIV positive men with **(B)** CD4+ T-cells >500/μL, **(C)** 200-499/μL and **(D)** <200/μL.
(PDF)

S6 Fig Mathematical model output comparison to HSV-2 episode duration. Ten mathematical model simulations (thin colored lines) in reference to empirical shedding data. Episode duration for **(A)** HIV negative, and HIV positive men with **(B)** CD4+ T-cells/μL >500, **(C)** 200–499 and **(D)** <200. Black horizontal bars represent 95% CI of episode duration if only episodes of known duration are analyzed. Red horizontal bars represent 95% CI of episode duration if all episodes are analyzed using interval censoring.
(PDF)

S1 Movie HSV-2 shedding simulation with parameters derived from HIV negative study participants. The upper left panel represents total cell-free HSV DNA copies / mL present over time. The upper right panel represents spatial spread of cell-free virus during the episode. The lower left panel represents CD8+ T-cell density within each region. The lower right panel indicates reproductive number within each region. Quantities are displayed according to a heat map adjacent to each spatial map. The reproductive number (R) represents the growth potential of virus in a given region and is inversely correlated with CD+ T-cell density: values less than 1 (log 10 R = 0) are grey or black and indicate no potential for extended viral spread, while values exceeding 1 indicate potential for prolonged viral growth.
(MP4)

S2 Movie HSV-2 shedding simulation with parameters derived from HIV positive study participants with CD4+ T-cell count < 200 / uL. The upper left panel represents total cell-free HSV DNA copies / mL present over time. The upper right panel represents spatial spread of cell-free virus during the episode. The lower left panel represents CD8+ T-cell density within each region. The lower right panel indicates reproductive number within each region. Quantities are displayed according to a heat map adjacent to each spatial map. The reproductive number (R) represents the growth potential of virus in a given region and is inversely correlated with CD+ T-cell density: values less than 1 (log 10 R = 0) are grey or black and indicate no potential for extended viral spread, while values exceeding 1 indicate potential for prolonged viral growth.
(MP4)
