# Supplementary figures and images for: Mathematical Modeling Predicts that Increased HSV-2 Shedding in HIV-1 Infected Persons Is Due to Poor Immunologic Control in Ganglia and Genital Mucosa
Source: PLoS One. 2016 Jun 10;11(6):e0155124. doi: 10.1371/journal.pone.0155124 (PMC4902308; doi:10.1371/journal.pone.0155124)

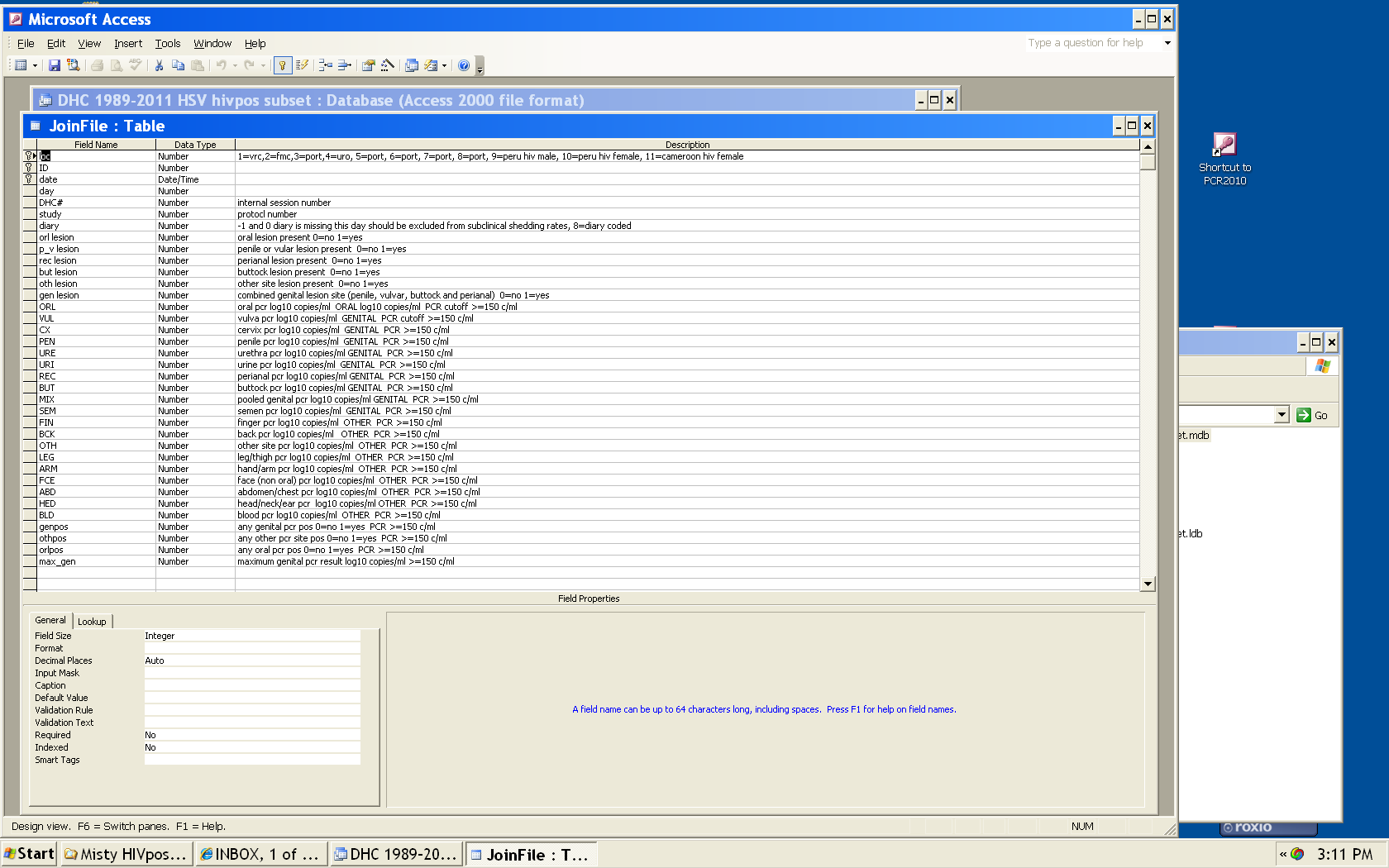

Supplement: S3 Data — (DOC) [file pone.0155124.s003.doc]

S1

a

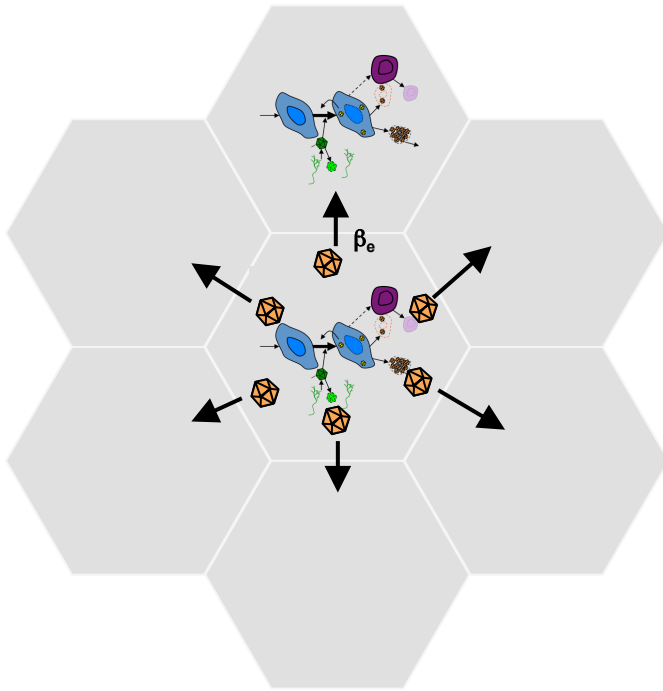

b

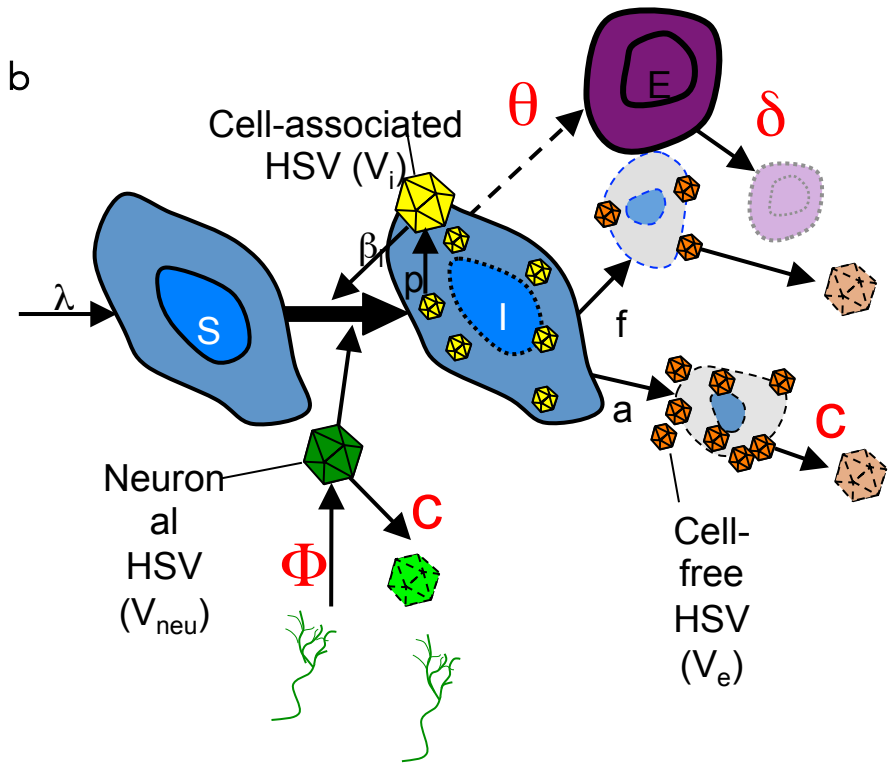

Supplement: S1 Fig — (A) Micro-regions are linked virally because cell-free HSV-2 can seed surrounding regions. (B) Schematic for HSV-2 infection within a single genital tract microenvironment. (PDF) [file pone.0155124.s004.pdf]

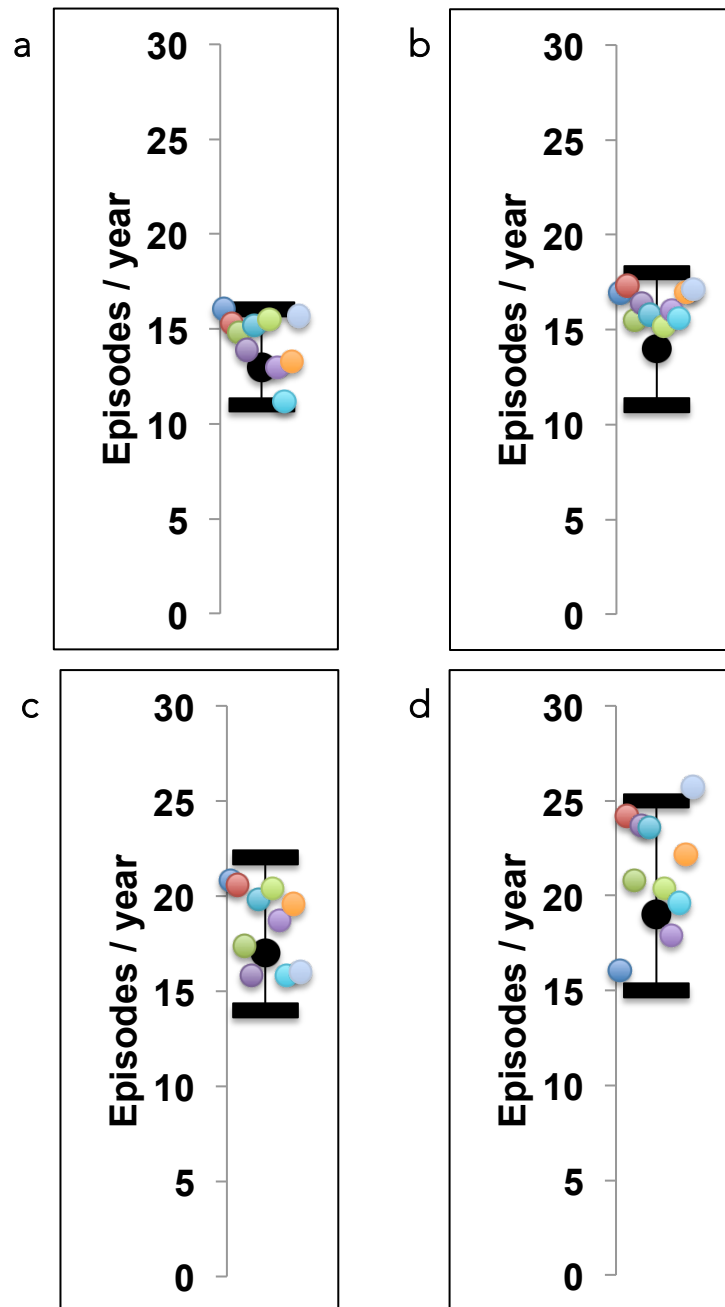

Supplement: S2 Fig — Ten mathematical model simulations of HSV-2 shedding (colored dots) in reference to empirical shedding data (median marked with black dot and 95% CI with black horizontal bars). Annualized episode rate for (A) HIV negative, and HIV positive men with (B) CD4+ T-cells >500/μL, (C) 200-499/μL and (D) <200/μL. (PDF) [file pone.0155124.s005.pdf]

S3

a

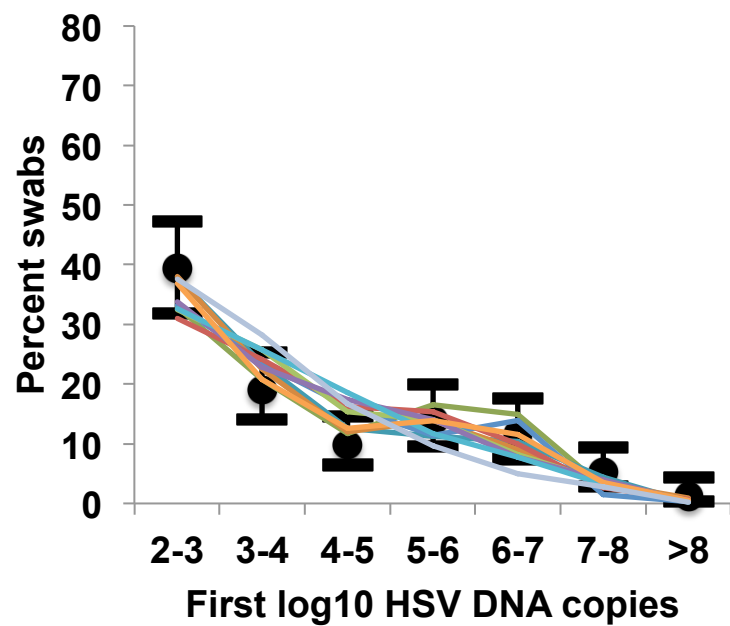

b

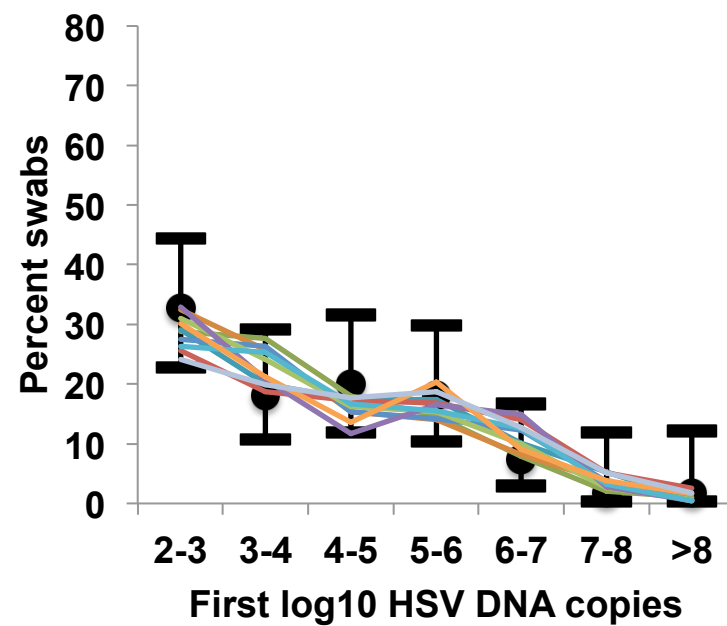

c

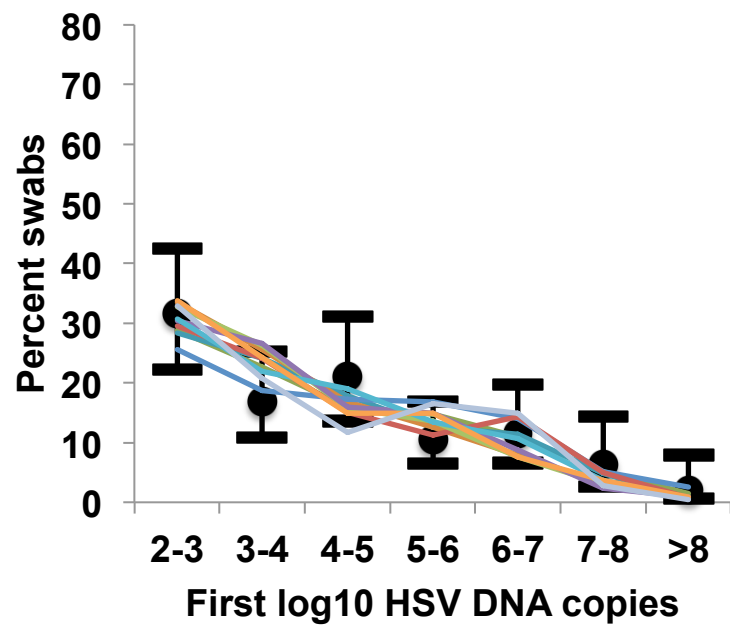

d

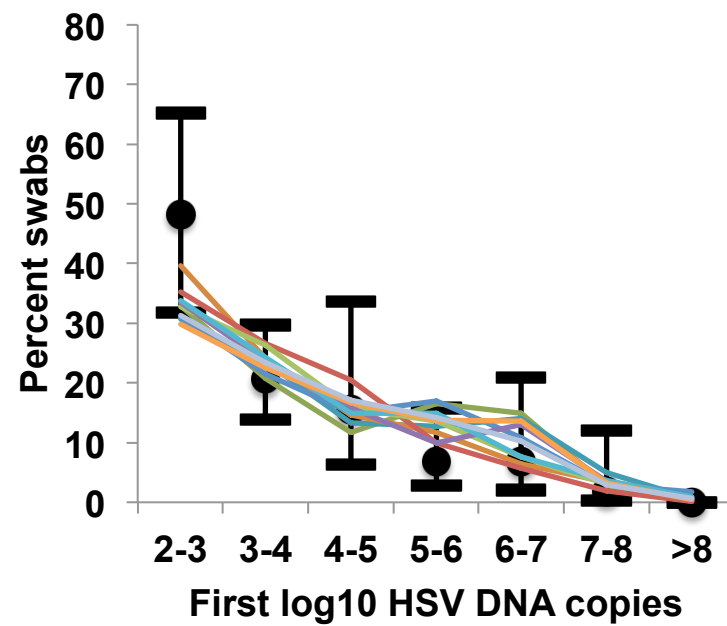

Supplement: S3 Fig — Ten mathematical model simulations (thin colored lines) in reference to empirical shedding data (median marked with black dot and 95% CI with black horizontal bars). First positive HSV DNA copy number per episode for (A) HIV negative, and HIV positive men with (B) CD4+ T-cells >500/μL, (C) 200-499/μL and (D) <200/μL. (PDF) [file pone.0155124.s006.pdf]

S4

a

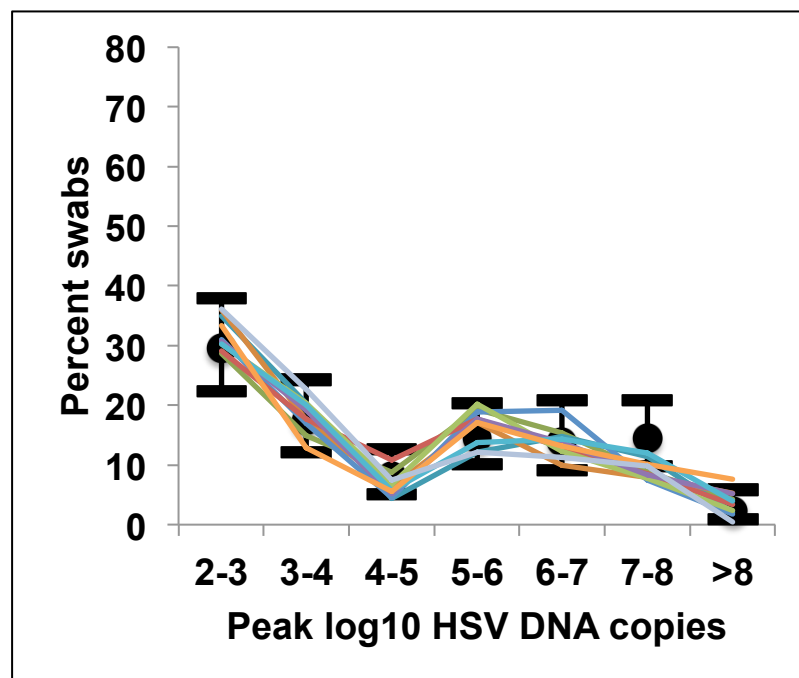

b

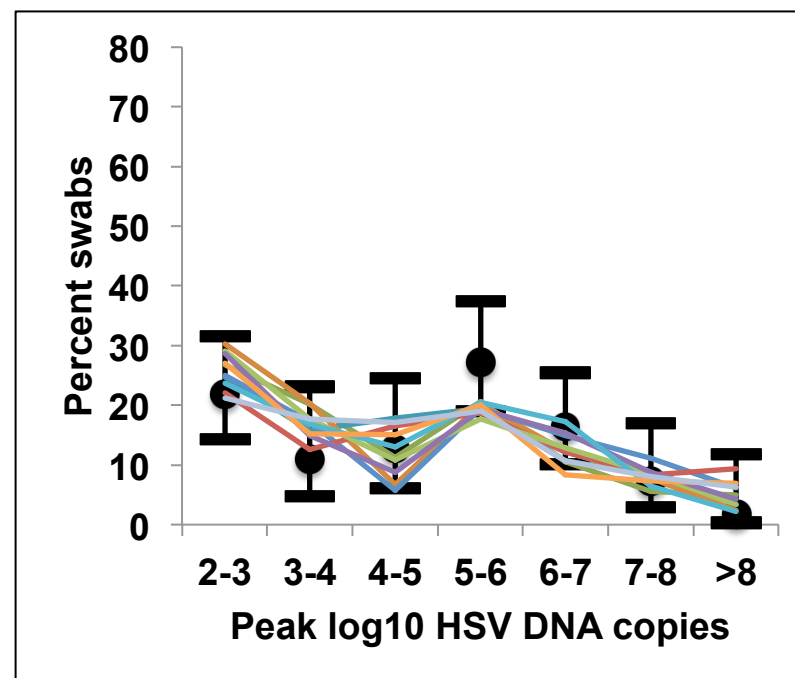

c

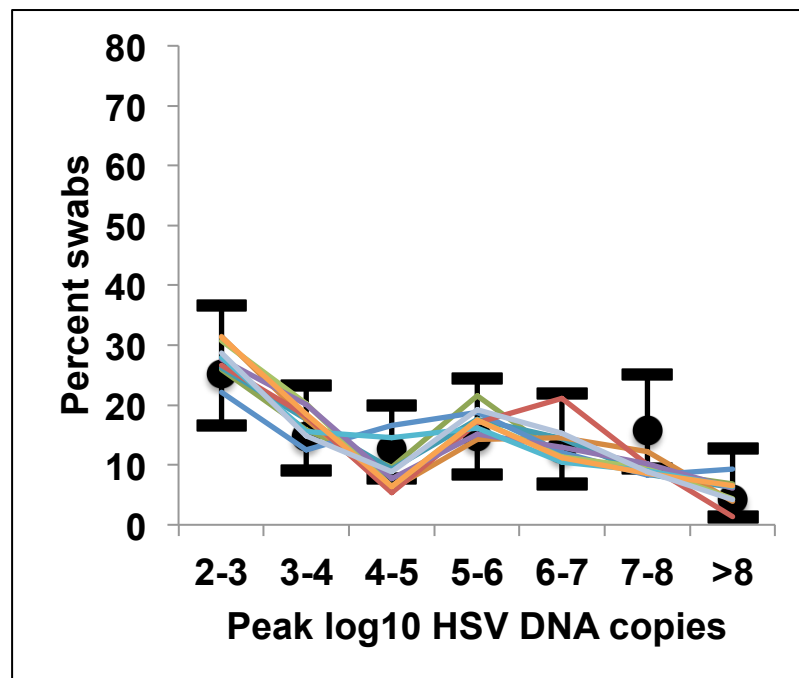

d

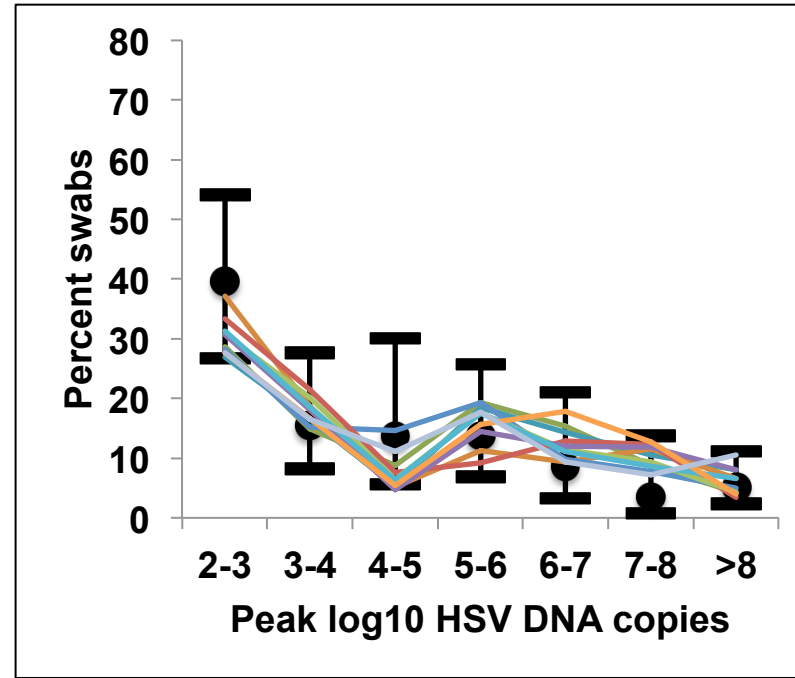

Supplement: S4 Fig — Ten mathematical model simulations (thin colored lines) in reference to empirical shedding data (median marked with black dot and 95% CI with black horizontal bars). Peak positive HSV DNA copy number per episode for (A) HIV negative, and HIV positive men with (B) CD4+ T-cells >500/μL, (C) 200-499/μL and (D) <200/μL. (PDF) [file pone.0155124.s007.pdf]

S5

a

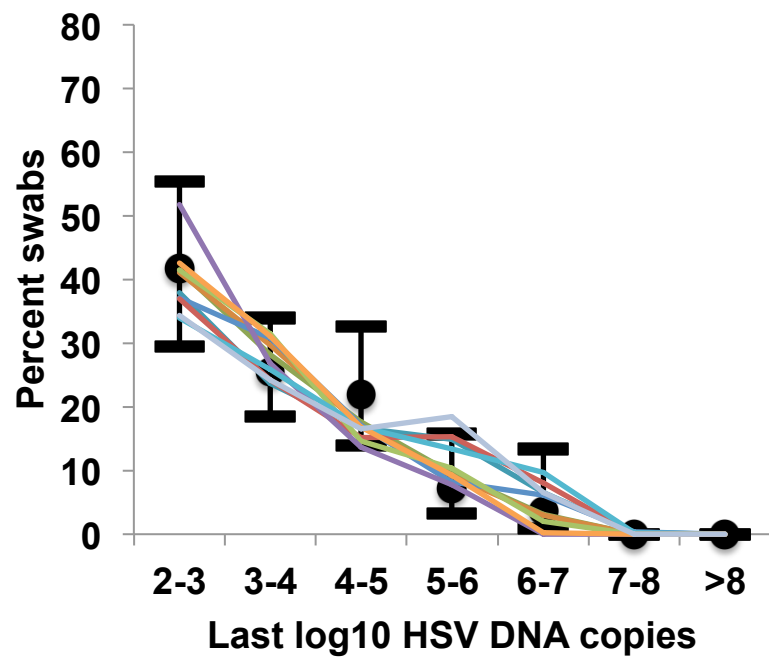

b

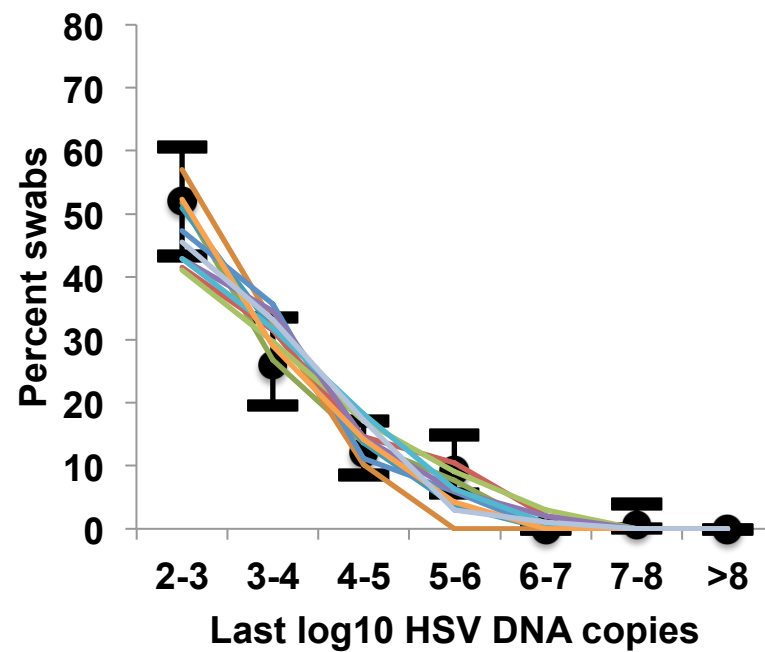

c

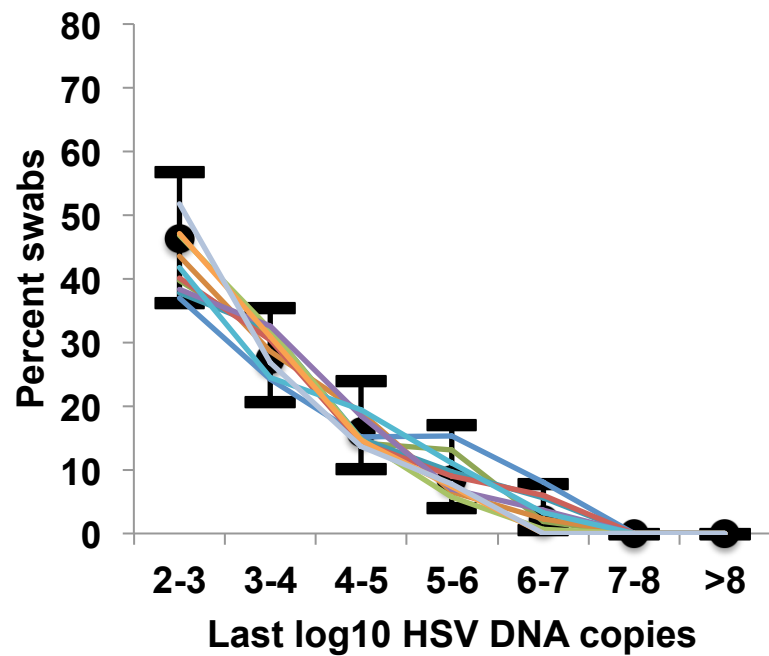

d

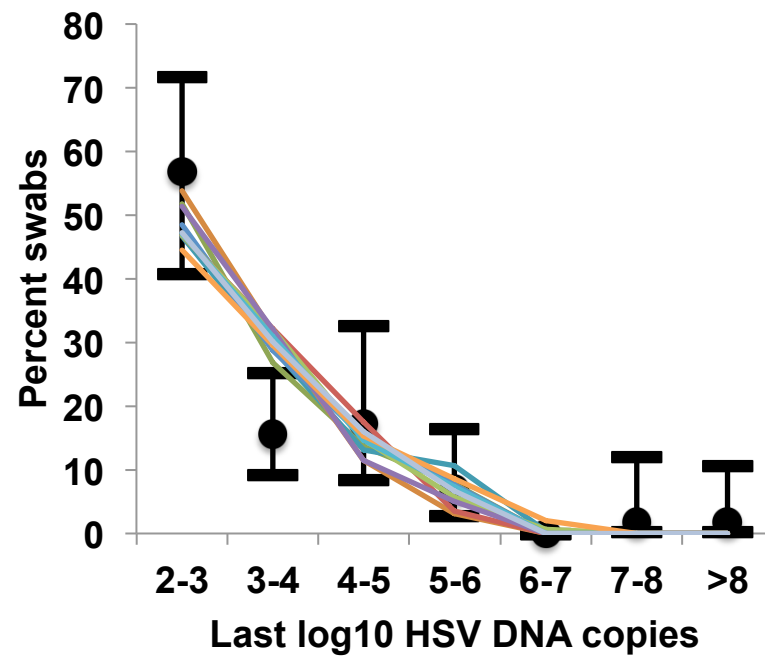

Supplement: S5 Fig — Ten mathematical model simulations (thin colored lines) in reference to empirical shedding data (median marked with black dot and 95% CI with black horizontal bars). Last positive HSV DNA copy number per episode for (A) HIV negative, and HIV positive men with (B) CD4+ T-cells >500/μL, (C) 200-499/μL and (D) <200/μL. (PDF) [file pone.0155124.s008.pdf]

S6

a

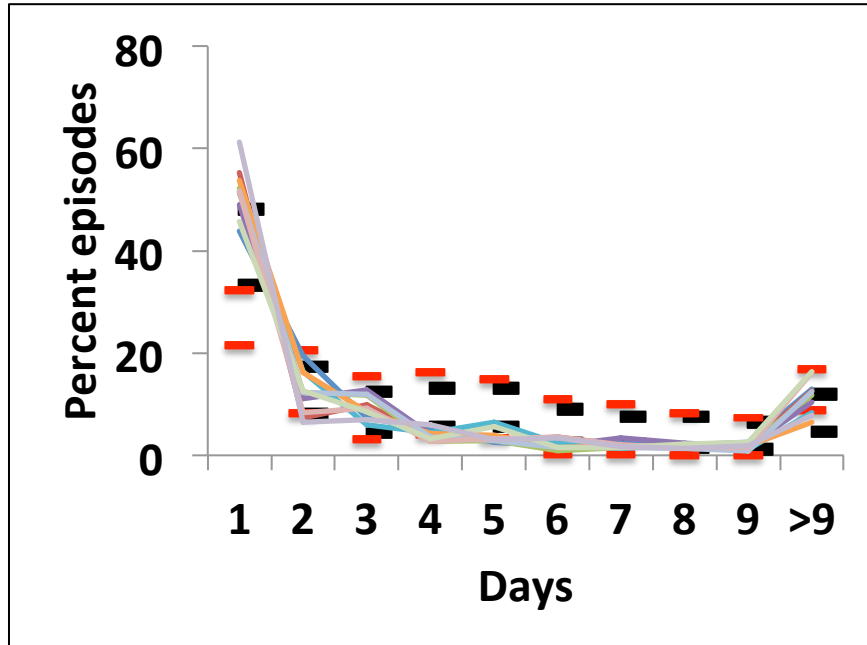

b

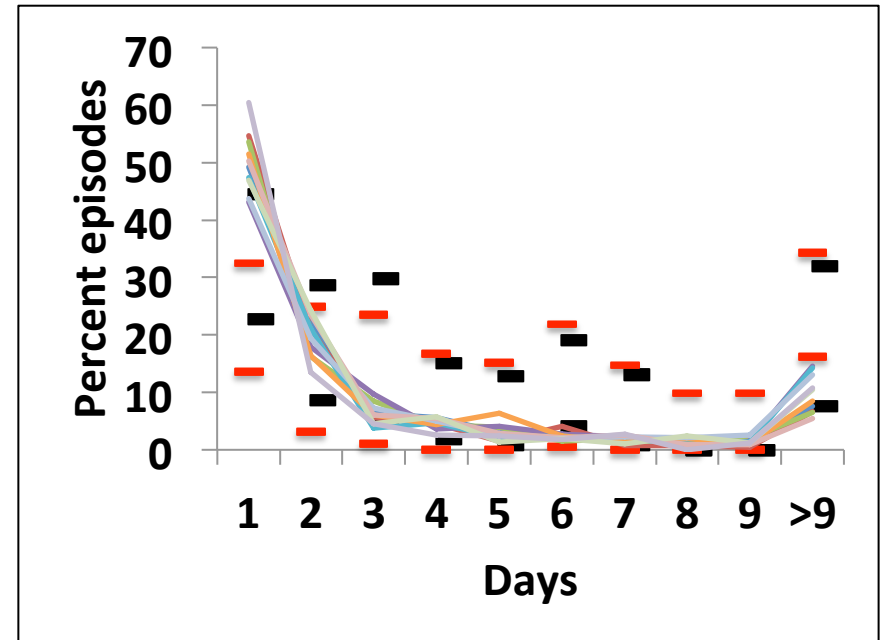

c

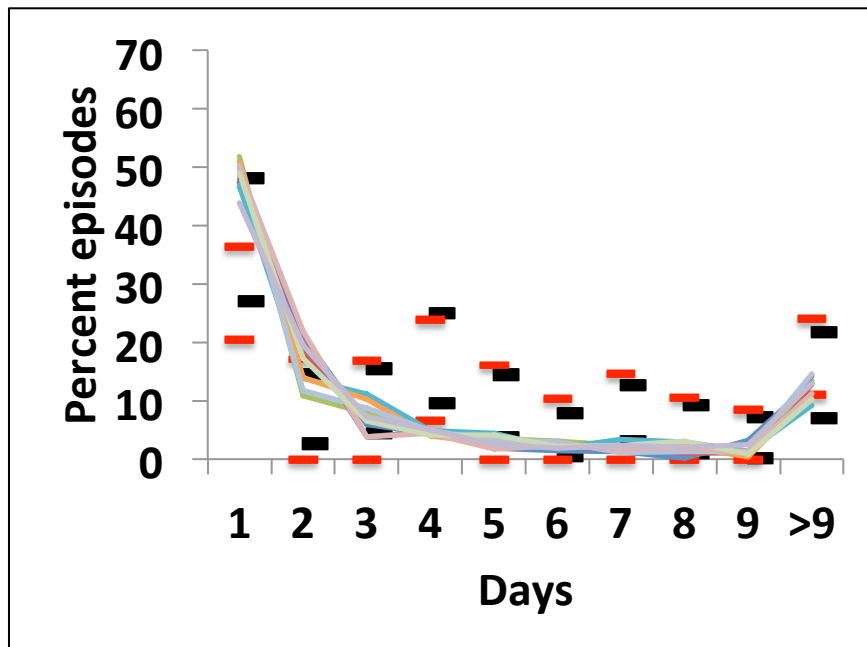

d

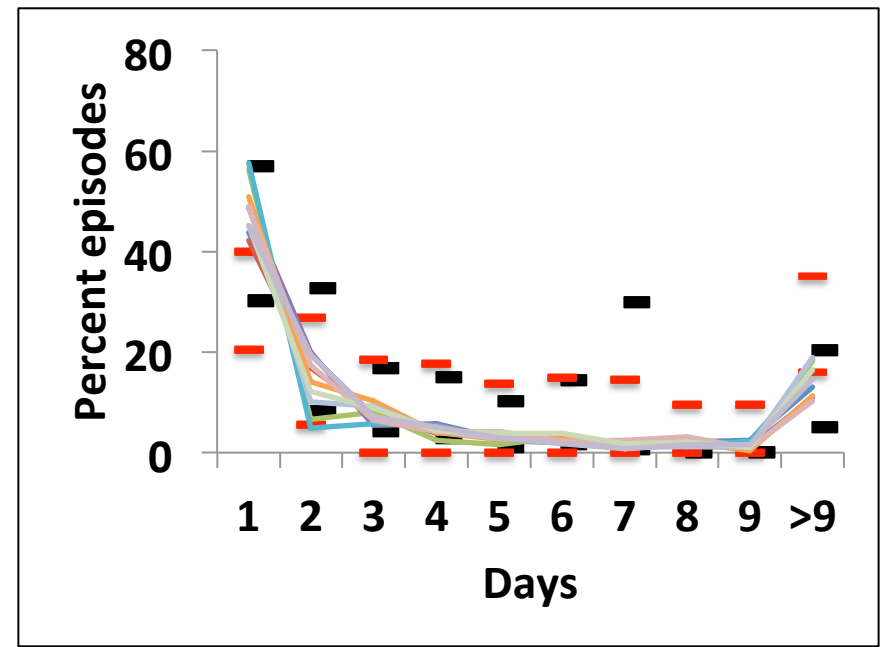

Supplement: S6 Fig — Ten mathematical model simulations (thin colored lines) in reference to empirical shedding data. Episode duration for (A) HIV negative, and HIV positive men with (B) CD4+ T-cells/μL >500, (C) 200–499 and (D) <200. Black horizontal bars represent 95% CI of episode duration if only episodes of known duration are analyzed. Red horizontal bars represent 95% CI of episode duration if all episodes are analyzed using interval censoring. (PDF) [file pone.0155124.s009.pdf]
